# Association between extended-release buprenorphine adherence and reduced healthcare costs among insured patients with opioid use disorder

**DOI:** 10.3389/fpubh.2026.1774410

**Published:** 2026-03-05

**Authors:** Michelle Jerry, Divya Venkat, Courtney Flynn, Anh Thu Tran, Maher Abdel-Sattar, William Mullen, Colleen Lane

**Affiliations:** 1 Research & Analytic Services, Merative, Ann Arbor, MI, United States; 2 Center for Inclusion Health, Allegheny Health Network, RIvER Clinic, Pittsburgh, PA, United States; 3 Real-World Evidence, Medical Affairs, Indivior Inc., North Chesterfield, VA, United States; 4 Addiction Medicine, Corewell Health West, Grand Rapids, MI, United States

**Keywords:** adherence, extended-release buprenorphine, healthcare cost, long-acting injectable, opioid use disorder, sublocade

## Abstract

**Purpose:**

To evaluate the relationship between adherence to extended-release buprenorphine (BUP-XR; SUBLOCADE®) and reduced healthcare utilization and medical costs among patients with opioid use disorder (OUD).

**Methods:**

This retrospective analysis of United States insurance claims included patients aged ≥18 years who initiated BUP-XR between 3/1/2019–12/31/2022 (index = earliest BUP-XR claim) in the Merative™ MarketScan® Commercial and Medicare Databases. Patients had continuous enrollment (medical, pharmacy, and behavioral benefits) for 12 months before (baseline period) and 12 months after (follow-up period) the index date. Proportion of days covered (PDC) was used to assess adherence to both BUP-XR and medications for OUD (MOUD) overall during the follow-up period. Patients were assigned to four mutually-exclusive groups: Group 1 (adherent [PDC ≥ 0.8] to BUP-XR treatment); Group 2 (non-adherent to BUP-XR but adherent to overall MOUD); Group 3 (non-adherent to overall MOUD and primarily treated with BUP-XR); Group 4 (non-adherent to overall MOUD and not primarily treated with BUP-XR). Descriptive and adjusted analyses compared total non-MOUD costs in the follow-up period of Group 1 (reference) to the other groups.

**Results:**

Of 661 patients, 24.7% were adherent to BUP-XR (Group 1) during the 12-month follow-up period (Group 2: 17.1%, Group 3: 39.9%, Group 4: 18.3%). Compared to other groups, patients adherent to BUP-XR (Group 1) had the lowest rates of inpatient, emergency department, and detoxification visits. Adjusted non-MOUD costs during the 12-month follow-up period were $35,761 (Group 1), $50,778 (Group 2), $29,599 (Group 3), and $44,739 (Group 4). Despite both groups having PDC ≥ 0.8, patients adherent to BUP-XR (Group 1) had $15,017 reduced costs per person compared to those adherent to overall MOUD (Group 2). The lower adjusted costs observed in Groups 3 and 4 reflected patient disengagement from care, characterized by substantially fewer outpatient services and higher acute care utilization.

**Conclusion:**

Sustained BUP-XR adherence reduced costly medical utilization compared to non-adherence to BUP-XR while maintaining adherence to overall MOUD.

## 1 Introduction

Opioid use disorder (OUD) is a chronic, relapsing brain disease resulting from the chronic use or misuse of prescribed or illicit opioids, including heroin and synthetic compounds like fentanyl. OUD is associated with substantial morbidity and mortality, including overdose (1). Among people aged 12 and older in 2024 in the United States (US), an estimated 1.7% or 4.8 million individuals were affected by OUD in the past year (2). The lethality of the opioid epidemic has been amplified by the rise of illicit, high-potency synthetic opioids such as fentanyl and its analogs (e.g., carfentanil) (3). Provisional US data from April 2025 showed that 87% of 48,422 opioid-involved overdose deaths reported in the previous 12 months involved synthetic opioids other than methadone (4). Expanded access to opioid overdose reversal agents has contributed to a decline in fatal opioid overdoses since 2023 (2, 5); however, overdoses remain a significant public health challenge.

The estimated economic burden of OUD has increased dramatically from nearly $1.5 trillion in 2020 (6) to $2.7 trillion in 2023 (7), reflecting costs attributable to fatal overdose, direct healthcare expenses, criminal justice, lost productivity, and reduced quality of life. Individuals with OUD are more likely to utilize more healthcare services and incur higher direct healthcare costs than individuals without OUD (8–11). For example, a claims-based analysis of commercially insured patients from 2012 to 2015 reported that individuals with OUD incurred an additional $14,810 in annual healthcare costs (9).

The Food and Drug Administration (FDA) in the US has approved several medications for OUD (MOUD), such as methadone, transmucosal buprenorphine (TM-BUP) alone or in combination with naloxone, and long-acting injectables (subcutaneous extended-release buprenorphine [BUP-XR] and intramuscular extended-release naltrexone [NTX-XR]) (1, 12). Despite being an effective component of OUD treatment, MOUD utilization remains low, with less than 1 in 5 patients receiving treatment. Among the 4.8 million people aged 12 or older in 2024 who experienced OUD in the past year, only 17.0% (or 818,000 people) received MOUD (2). In addition, MOUD retention is limited. Among 130,300 discharges from outpatient substance use treatment facilities in 2017, 64% discontinued MOUD within 6 months (13).

Adherence to MOUD is critical for improving clinical outcomes and reducing healthcare expenditures among patients with OUD. Prior studies have established the relationship between adherence to TM-BUP and lower healthcare costs, but the relationship between monthly injectable BUP-XR adherence and healthcare costs is not well reported. The present study addresses this gap by evaluating the relationship between BUP-XR adherence and healthcare utilization and costs among patients with OUD in the US, using administrative claims data from a large commercially insured population. The analysis was also conducted separately for patients with Medicaid insurance, but due to the high proportion of Medicaid patients in capitated arrangements (i.e., managed care), cost reporting for the Medicaid population was not reliable/representative. The real-world evidence generated from this analysis can inform public health strategies and payer policies on interventions with effective, long-acting OUD treatments.

## 2 Materials and methods

### 2.1 Data source and study design

This was a retrospective, observational study of longitudinal administrative claims data from the Merative™ MarketScan® Commercial, Medicare, and Multi-state Medicaid Databases. These databases contain fully adjudicated claims for inpatient, outpatient medical, and outpatient pharmacy services for individuals with employer-based commercial health plans, Medicare Advantage, and Medicare Supplemental (Commercial/Medicare sample), as well as Medicaid enrollees (Medicaid sample). All database records were de-identified and fully compliant with US patient confidentiality requirements, including the Health Insurance Portability and Accountability Act of 1996. Because this study only used de-identified patient records and did not involve the collection, use, or transmittal of individually identifiable data, Institutional Review Board approval was not deemed necessary.

### 2.2 Study population

Patients were included in the analysis if they had ≥1 claim for BUP-XR between March 1, 2019 and December 31, 2022, with the earliest BUP-XR claim date serving as the index date (i.e., BUP-XR initiation). Because no other long-acting injectable buprenorphine products were available during the patient selection period, the BUP-XR product was only SUBLOCADE®. Patients were required to be ≥18 years old on the index date, to have continuous enrollment with medical and pharmacy benefits for 12 months prior to the index date (pre-index period) and 12 months following the index date (post-index period; includes the index date), and to have no claims for NTX-XR on or within 30 days preceding the index date. To ensure that patients were starting a new treatment episode with BUP-XR on the index date, patients were excluded if they had a claim for BUP-XR during the pre-index period. Additionally, patients whose substance abuse/mental health data was known to be missing from the database were excluded. Finally, patients who were dual-eligible for both Medicaid and Medicare were excluded from the Medicaid sample, as services covered fully by Medicare may not be sent to the Medicaid insurance provider and thus may not appear in the Medicaid database.

There has been an increasing shift toward capitated payment models in US Medicaid programs (i.e., managed care). While claims data for patients in Medicaid managed care arrangements accurately reflect the services received and payments for those services, the reported costs are underestimated due to capitation and thus are not directly comparable to costs captured for Medicaid patients in fee-for-service (FFS) arrangements or commercially insured patients. Because of this cost reporting limitation, this study was conducted separately for Commercial/Medicare and Medicaid samples. Findings from the Commercial/Medicare analysis are reported in the body of the manuscript, while the Medicaid analysis is presented in the Supplementary material B. While cost reporting was conducted for the subset of Medicaid patients who were in FFS arrangements for the full study period (approximately 10% of the Medicaid sample), these results are not presented due to small sample size and because they are not representative of overall Medicaid.

### 2.3 Adherence

Adherence to BUP-XR treatment and overall MOUD treatment were measured based on proportion of days covered (PDC) during the 12-month post-index period. Overall MOUD treatment included all BUP- and NTX-based MOUD (BUP-XR, TM-BUP, and NTX-XR). US federal regulations mandate that methadone for OUD be dispensed only through specialized Opioid Treatment Programs (OTPs) on an almost daily basis rather than via standard pharmacies or office-based settings. Consequently, methadone was not considered in this analysis because it cannot be accurately identified in administrative claims data. For both formulations of MOUD (injectable and non-injectable), PDC was calculated as the total days of medication coverage divided by the duration of the reporting period. For TM-BUP, the days supply information on the outpatient pharmacy claim was used to assign days of medication coverage, while BUP-XR and NTX-XR were each assigned 30 days of coverage (consistent with a monthly injection). PDC categories included: <0.2, 0.2 to <0.4, 0.4 to <0.6, 0.6 to <0.8, and ≥0.8. Additionally, BUP-XR PDC and overall MOUD PDC were assessed during the first and second halves of the post-index period to evaluate changes in adherence over time.

Based on their BUP-XR and overall MOUD PDC values during the 12-month post-index period, patients were categorized into one of four reporting groups: Group 1 (adherent to BUP-XR treatment); Group 2 (non-adherent to BUP-XR but adherent to overall MOUD); Group 3 (non-adherent to overall MOUD and primarily treated with BUP-XR); Group 4 (non-adherent to overall MOUD and not primarily treated with BUP-XR; Table 1). Consistent with prior OUD studies, patients were classified as adherent if their PDC was ≥0.8. Patients were classified as being primarily treated with BUP-XR if their BUP-XR PDC and overall MOUD PDC were both in the same adherence range (e.g., if a patient has both BUP-XR PDC and overall MOUD PDC in the 0.4 to <0.6 range then we deduce that BUP-XR was the primary medication utilized, with minimal to no use of other MOUD such as TM-BUP or NTX-XR).

**Table 1:** Definition of PDC groups.

**Overall MOUD PDC**	**BUP-XR PDC**				
	<0.2	0.2 to <0.4	0.4 to <0.6	0.6 to <0.8	≥0.8
<0.2					
0.2 to <0.4					
0.4 to <0.6	Group 4		Group 3		
0.6 to <0.8					
≥0.8		Group 2			Group 1
**Adherence group definitions**					
Group 1	Adherent to BUP-XR treatment				
Group 2	Non-adherent to BUP-XR but adherent to overall MOUD				
Group 3	Non-adherent to overall MOUD and primarily treated with BUP-XR				
Group 4	Non-adherent to overall MOUD and not primarily treated with BUP-XR				

BUP-XR, extended-release buprenorphine; MOUD, medication for opioid use disorder; NTX-XR, extended-release naltrexone; PDC, proportion of days covered; TM-BUP, transmucosal buprenorphine. Adherence was measured by PDC for both BUP-XR and overall MOUD during the 12-month post-index period, categorized as: PDC<0.2, 0.2 ≤ PDC<0.4, 0.4 ≤ PDC<0.6, 0.6 ≤ PDC<0.8, and PDC≥0.8. Overall MOUD included buprenorphine- or naltrexone-based MOUD (BUP-XR, TM-BUP, and NTX-XR). Patients were classified as adherent if they had PDC≥0.8. Patients were classified as being primarily treated with BUP-XR if their BUP-XR PDC and their overall MOUD PDC were both in the same adherence range (e.g., if a patient has both BUP-XR PDC and overall MOUD PDC in the 0.4 to <0.6 range then we deduce that BUP-XR was the primary medication utilized, with minimal to no use of other MOUD such as TM-BUP or NTX-XR). PDC groups are defined by the combination of overall MOUD and BUP-XR PDC categories. Group assignments are color-coded as follows: Group 1 (green), Group 2 (yellow), Group 3 (blue), and Group 4 (red). Grey cells indicate combinations of PDC categories that are not possible based on the group definition.

Though PDC ≥ 0.8 is a commonly accepted definition of adherence in claims-based studies, a lower threshold may align better with real-world clinical observation (14–17). Previous MOUD adherence studies have found that patients with a PDC of 0.6 to <0.8 may experience similar decreases in relapse and healthcare costs compared to patients with PDC ≥ 0.8, suggesting that it may be appropriate to consider interventions that effectively raise adherence to 0.6 or above as successful, even if the intervention is not able to move the patient to the optimal level of adherence of 0.8 (15, 16). To accommodate this clinical nuance, a sensitivity analysis was performed where the PDC groups were re-assigned using a less stringent threshold for adherence (PDC ≥ 0.6) to account for patients who may occasionally experience delays in their BUP-XR dosing beyond the recommended 4-week interval due to transportation difficulties, insurance coverage gaps, scheduling conflicts, or other access challenges (Supplementary Table 1). Supplementary material A includes all descriptive and adjusted analyses for the sensitivity analysis.

### 2.4 Measures

#### 2.4.1 Healthcare utilization and costs

All-cause utilization and costs were evaluated during the 12-month post-index period across four mutually exclusive service categories: inpatient admissions (excluding detoxification), outpatient services (excluding detoxification), detoxification, and outpatient pharmacy. Detoxification was differentiated from other inpatient and outpatient services based on diagnosis related group (DRG), revenue (REV), and procedure codes indicative of detoxification. Outpatient services were sub-categorized into emergency department (ED) visits, office visits, and other outpatient services (e.g., imaging, laboratory). Utilization and costs for MOUD treatment, urine drug screens, and psychosocial therapy were also reported separately.

Costs were derived from relevant claims, capturing both patient responsibility (e.g., deductible, copay or coinsurance) and health plan payments. Detoxification costs comprised the full cost of inpatient admissions with a detoxification code as well as detoxification-related outpatient claims. Total costs were calculated as the sum of inpatient, outpatient, detoxification, and outpatient pharmacy costs. MOUD costs were determined based on outpatient pharmacy-dispensed drugs and outpatient drug administrations (i.e., costs from claims with relevant NDC and HCPCS codes), while non-MOUD costs were calculated as total costs minus MOUD costs. All costs were inflated to 2023 US dollars using the medical component of the Consumer Price Index (18).

#### 2.4.2 Other variables

Demographics were recorded as of the index date and included: age, gender, population density, payer, insurance plan type, and index year. Baseline clinical characteristics were reported during the 12-month pre-index period plus the index date and included: Charlson Comorbidity Index (CCI) score, conditions/comorbidities, concomitant medication use, and MOUD use. CCI and conditions/comorbidities (OUD, alcohol use disorder, substance use disorder [other than opioids/alcohol], schizophrenia, depression/bipolar disorder, generalized anxiety disorder, HIV/AIDS, hepatitis B or C, pregnancy, endocarditis, skin and soft tissue infections, and chronic pain conditions) were determined based non-diagnostic inpatient or outpatient claims with a diagnosis. Concomitant medications (narcotic pain medications, benzodiazepines, sedative/hypnotics, antidepressants, and antipsychotics) and baseline MOUD use were determined based on outpatient pharmacy-dispensed drugs and outpatient drug administrations. Baseline MOUD measures included: proportion of patients with use of each individual MOUD (TM-BUP, BUP-XR, and NTX-XR) and PDC for overall MOUD (TM-BUP, BUP-XR, and NTX-XR combined). Additionally, baseline TM-BUP use during the 3-month pre-index period, including proportion of patients with use and PDC, were reported.

### 2.5 Statistical analyses

Reporting was conducted separately for the Commercial/Medicare sample and Medicaid sample (see Supplementary material B). Categorical variables were summarized by the count and percentage in each category, while continuous variables were summarized by means and standard deviations (SD). Results were presented for all BUP-XR treated patients (overall) and for the four PDC groups. Group 1 (adherent to BUP-XR treatment) served as the reference for all statistical comparisons. Statistical significance was assessed using Chi-square or Fisher’s exact tests for categorical variables and Student’s t-tests for continuous variables. The threshold for statistical significance was set *a priori* at *p* < 0.05.

For the Commercial/Medicare sample, multivariable modeling was used to adjust for differences in baseline characteristics among PDC groups that may be contributing to observed differences in mean non-MOUD costs during the 12 months after initiation of BUP-XR. One generalized linear model with log link and gamma error distribution was used to model the cost ratios for each group relative to Group 1. Aside from PDC group, the following covariates were included in the model: baseline demographics recorded on the index date (age, sex, population density [urban vs. rural]), baseline comorbidities during the 12-month pre-index period plus the index date (CCI, alcohol use disorder, other non-opioid/alcohol substance use disorder, skin and soft tissue infections), and baseline medication use (antidepressant/antipsychotic use during the 12-month pre-index period plus the index date, TM-BUP adherence [PDC ≥ 0.6 vs. PDC < 0.6 or no use] during the 3-month pre-index period). Population density was included to address potential differences in MOUD access related to geographic region. Conditions and CCI score were included to account for differences in patient comorbidity profile and OUD complexity/severity. Baseline medication use was included to address differences in prior treatment engagement. Based on the model output, predicted mean costs for each PDC group were derived using recycled predictions, whereby the entire sample was hypothetically assigned to each group to estimate the corresponding adjusted mean cost (e.g., if all patients were in Group 4, what would the predicted mean cost be?). A significance level of *p* < 0.05 was also pre-defined for the adjusted analysis.

## 3 Results

### 3.1 Patient selection and PDC groups

**Figure 1:**
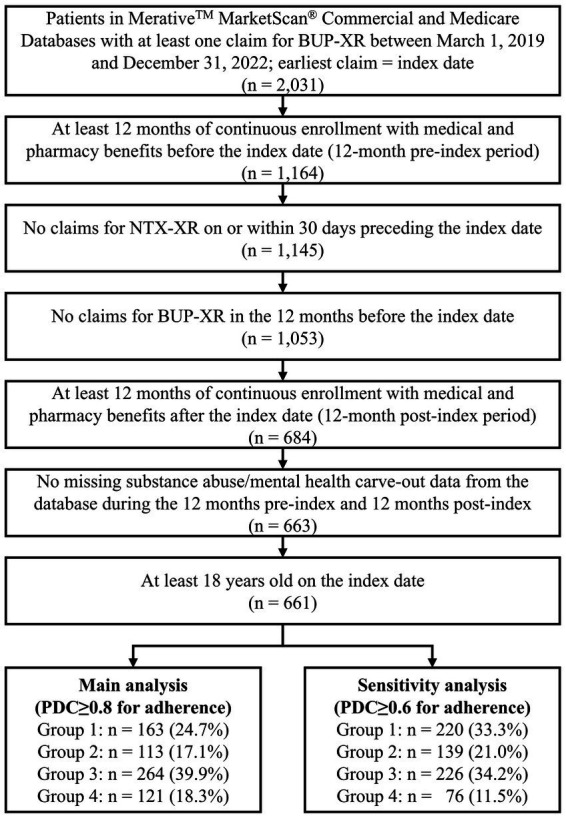
Patient attrition flow chart: commercial/Medicare patients. BUP-XR, extended-release buprenorphine; NTX-XR, extended-release naltrexone; PDC, proportion of days covered.

A total of 661 Commercial/Medicare patients initiating BUP-XR treatment met the patient selection criteria. About one quarter (24.7%) of those patients were adherent to BUP-XR (Group 1) during the 12-month follow-up compared to 17.1% (Group 2), 39.9% (Group 3), and 18.3% (Group 4; Figure 1). When defining the groups using the alternative PDC ≥ 0.6 threshold that may align better with real-world OUD clinical practice, the proportion of patients who were adherent to BUP-XR increased from 24.7 to 33.3%.

### 3.2 Patient characteristics

The mean age among included OUD patients was 37.5 years, with 67.6% male and 84.7% residing in urban areas (Table 2). Within this population, alcohol use disorder affected 22.1% of patients, with more common diagnoses including non-alcohol/non-opioid substance use disorders (52.7%), generalized anxiety disorder (52.0%), and depression/bipolar disorder (46.9%). Over two-thirds of patients (67.9%) were treated with antidepressants or antipsychotics in the 12 months prior to initiating BUP-XR. Additionally, nearly all patients (94.1%) had used some form of MOUD in the 12-month pre-index period, with 89.4% using TM-BUP in the 3 months prior to BUP-XR initiation.

**Table 2:** Demographic and clinical characteristics: commercial/Medicare patients.

Baseline demographics and clinical characteristics	Commercial/Medicare							
	All patients	Group 1	Group 2	*p*-value^a^	Group 3	*p*-value^a^	Group 4	*p*-value^a^
	(*n* = 661)	(*n* = 163)	(*n* = 113)		(*n* = 264)		(*n* = 121)	
Demographic characteristics (on index)								
Age, mean (SD)	37.5 (11.3)	40.0 (10.7)	40.8 (10.8)	0.546	36.6 (11.3)	0.002	33.3 (11.2)	<0.001
Age category, n (%)				0.535		0.080		<0.001
18–34	274 (41.5%)	53 (32.5%)	34 (30.1%)		114 (43.2%)		73 (60.3%)	
35–44	213 (32.2%)	53 (32.5%)	43 (38.1%)		87 (33.0%)		30 (24.8%)	
45–54	113 (17.1%)	39 (23.9%)	21 (18.6%)		43 (16.3%)		10 (8.3%)	
55–64	59 (8.9%)	18 (11.0%)	14 (12.4%)		19 (7.2%)		8 (6.6%)	
65–74	2 (0.3%)	0 (0.0%)	1 (0.9%)		1 (0.4%)		0 (0.0%)	
Sex, n (%)				0.490		0.436		0.202
Male	447 (67.6%)	109 (66.9%)	80 (70.8%)		186 (70.5%)		72 (59.5%)	
Female	214 (32.4%)	54 (33.1%)	33 (29.2%)		78 (29.6%)		49 (40.5%)	
Population density, n (%)				0.651		0.486		0.617
Urban	560 (84.7%)	136 (83.4%)	90 (79.7%)		228 (86.4%)		106 (87.6%)	
Rural	96 (14.5%)	25 (15.3%)	22 (19.5%)		35 (13.3%)		14 (11.6%)	
Unknown	5 (0.8%)	2 (1.2%)	1 (0.9%)		1 (0.4%)		1 (0.8%)	
Insurance plan type, n (%)				0.464		0.356		0.872
Comprehensive/Indemnity	17 (2.6%)	4 (2.5%)	5 (4.4%)		2 (0.8%)		6 (5.0%)	
EPO/PPO	373 (56.4%)	93 (57.1%)	69 (61.1%)		146 (55.3%)		65 (53.7%)	
POS/POS with capitation	71 (10.7%)	22 (13.5%)	9 (8.0%)		26 (9.9%)		14 (11.6%)	
HMO	77 (11.7%)	15 (9.2%)	13 (11.5%)		36 (13.6%)		13 (10.7%)	
CDHP/HDHP	116 (17.6%)	28 (17.2%)	15 (13.3%)		51 (19.3%)		22 (18.2%)	
Other/Unknown	7 (1.1%)	1 (0.6%)	2 (1.8%)		3 (1.1%)		1 (0.8%)	
Index year, n (%)				0.906		0.389		0.378
2019	119 (18.0%)	35 (21.5%)	25 (22.1%)		41 (15.5%)		18 (14.9%)	
2020	135 (20.4%)	36 (22.1%)	21 (18.6%)		55 (20.8%)		23 (19.0%)	
2021	163 (24.7%)	36 (22.1%)	25 (22.1%)		69 (26.1%)		33 (27.3%)	
2022	244 (36.9%)	56 (34.4%)	42 (37.2%)		99 (37.5%)		47 (38.8%)	
Baseline clinical characteristics (during 12-month pre-index period plus index date)								
Charlson Comorbidity Index, mean (SD)	0.4 (0.8)	0.3 (0.7)	0.3 (0.6)	0.795	0.3 (0.8)	0.981	0.5 (1.2)	0.193
Clinical conditions^b^, n (%)								
Opioid use disorder^c^	585 (88.5%)	145 (89.0%)	103 (91.2%)	0.686	227 (86.0%)	0.457	110 (90.9%)	0.693
Alcohol use disorder^c^	146 (22.1%)	30 (18.4%)	24 (21.2%)	0.644	53 (20.1%)	0.707	39 (32.2%)	0.008
Substance use disorder (other than opioids/alcohol)^c^	348 (52.7%)	86 (52.8%)	54 (47.8%)	0.463	133 (50.4%)	0.690	75 (62.0%)	0.146
Schizophrenia	5 (0.8%)	1 (0.6%)	0 (0.0%)	1.000	1 (0.4%)	1.000	3 (2.5%)	0.316
Depression/bipolar disorder	310 (46.9%)	76 (46.6%)	50 (44.3%)	0.714	107 (40.5%)	0.228	77 (63.6%)	0.006
Generalized anxiety disorder	344 (52.0%)	79 (48.5%)	55 (48.7%)	1.000	128 (48.5%)	1.000	82 (67.8%)	0.002
HIV/AIDS	2 (0.3%)	0 (0.0%)	0 (0.0%)	1.000	1 (0.4%)	1.000	1 (0.8%)	0.426
Hepatitis B or C	37 (5.6%)	8 (4.9%)	2 (1.8%)	0.207	16 (6.1%)	0.672	11 (9.1%)	0.229
Pregnancy	19 (2.9%)	2 (1.2%)	1 (0.9%)	1.000	8 (3.0%)	0.330	8 (6.6%)	0.021
Endocarditis	1 (0.2%)	0 (0.0%)	0 (0.0%)	1.000	1 (0.4%)	1.000	0 (0.0%)	1.000
Skin and soft tissue infections	61 (9.2%)	12 (7.4%)	7 (6.2%)	0.812	22 (8.3%)	0.854	20 (16.5%)	0.022
Chronic pain conditions	261 (39.5%)	68 (41.7%)	49 (43.4%)	0.805	100 (37.9%)	0.476	44 (36.4%)	0.391
Concomitant medications, n (%)								
Narcotic pain medications	132 (20.0%)	33 (20.3%)	27 (23.9%)	0.553	49 (18.6%)	0.705	23 (19.0%)	0.880
Benzodiazepines	194 (29.4%)	40 (24.5%)	33 (29.2%)	0.407	71 (26.9%)	0.650	50 (41.3%)	0.003
Sedative/hypnotics	244 (36.9%)	43 (26.4%)	41 (36.3%)	0.085	99 (37.5%)	0.020	61 (50.4%)	<0.001
Antidepressants/antipsychotics	449 (67.9%)	115 (70.6%)	75 (66.4%)	0.509	162 (61.4%)	0.060	97 (80.2%)	0.074
Antidepressants	426 (64.5%)	110 (67.5%)	74 (65.5%)	0.795	156 (59.1%)	0.100	86 (71.1%)	0.604
Antipsychotics	195 (29.5%)	42 (25.8%)	26 (23.0%)	0.671	70 (26.5%)	0.910	57 (47.1%)	<0.001
Baseline MOUD use (during 12-month pre-index period)								
Any use of buprenorphine- or naltrexone-based MOUD^d^, n (%)	622 (94.1%)	154 (94.5%)	113 (100.0%)	0.012	237 (89.8%)	0.107	118 (97.5%)	0.247
TM-BUP	621 (94.0%)	154 (94.5%)	113 (100.0%)	0.012	236 (89.4%)	0.078	118 (97.5%)	0.247
NTX-XR	20 (3.0%)	4 (2.5%)	0 (0.0%)	0.147	8 (3.0%)	1.000	8 (6.6%)	0.133
PDC^e^ for buprenorphine- or naltrexone-based MOUD, mean (SD)	0.6 (0.4)	0.7 (0.3)	0.7 (0.3)	0.267	0.5 (0.4)	<0.001	0.5 (0.3)	<0.001
Baseline TM-BUP use (during 3-month pre-index period), n (%)				0.164		<0.001		<0.001
PDC^e^ ≥0.6	400 (60.5%)	117 (71.8%)	88 (77.9%)		132 (50.0%)		63 (52.1%)	
PDC^e^ <0.6	191 (28.9%)	33 (20.3%)	22 (19.5%)		87 (33.0%)		49 (40.5%)	
No use of TM-BUP	70 (10.6%)	13 (8.0%)	3 (2.7%)		45 (17.1%)		9 (7.4%)	

AIDS, acquired immunodeficiency syndrome; CDHP, consumer-driven health plan; EPO, exclusive provider organization; HDHP, high-deductible health plan; HIV, human immunodeficiency virus; HMO, health maintenance organization; MOUD, medication for opioid use disorder; NTX-XR, extended-release naltrexone; PDC, proportion of days covered; POS, point of service; PPO, preferred provider organization; SD, standard deviation; TM-BUP, transmucosal buprenorphine.

a Based on comparison with Group 1.

b Based on non-diagnostic claims (inpatient or outpatient) with a diagnosis for the given condition.

c All substance use disorders include codes for substance abuse and dependence (as applicable).

d Based on the patient selection criteria, no patients received extended-release buprenorphine during the 12-month pre-index period or NTX-XR during the 30-day pre-index period.

e Calculated as the total days of possession of the medication divided by the length of the reporting period (365 or 90 days). The total days with possession of the medication during the reporting period was calculated regardless of gaps in therapy. For TM-BUP, overlapping days’ supply were appended to the total days’ supply.

Within the PDC groups, Groups 1 and 2 were older (mean age: 40.0 and 40.8 years, respectively), whereas Groups 3 and 4 were younger (mean age: 36.6 and 33.3 years, respectively), with Group 4 being the youngest. All groups were predominantly male, with the highest proportion of males in Group 2 (70.8%) followed by Group 3 (70.5%), Group 1 (66.9%), and Group 2 (59.5%). Group 4 had the highest clinical burden, with higher prevalence of many comorbidities (such as alcohol use disorder [32.2%], other substance use disorder [62.0%], depression/bipolar disorder [63.6%], generalized anxiety disorder [67.8%], skin and soft tissue infections [16.5%], and others) and concomitant medications (anti-depressants/antipsychotics [80.2%], and others) compared to other groups. Nearly all patients across groups had used some form of MOUD in the 12-month pre-index period, with baseline TM-BUP use in the 3 months prior to BUP-XR initiation being the highest in Group 2 (97.3%) followed by Group 4 (92.6%), Group 1 (92.0%), and Group 3 (82.9%).

### 3.3 Adherence

Over the 12-month post-index period, Group 1 consistently exceeded the PDC ≥ 0.8 adherence threshold, with PDC > 0.9 in both the first and second half of follow-up (Table 3). Group 2 also maintained an overall MOUD PDC > 0.9 during both six-month periods, though their adherence to BUP-XR was lower and declined across the two periods (PDC decreasing from 0.47 to 0.17). In contrast, Groups 3 and 4 showed substantial declines in both BUP-XR and overall MOUD adherence, with mean overall MOUD PDC decreasing across the two periods from 0.61 to 0.13 in Group 3 and from 0.66 to 0.33 in Group 4 (Table 3). These patterns suggest sustained engagement with MOUD treatment among patients in Groups 1 and 2, but low treatment engagement, especially in the second half of the follow-up period, among patients in Groups 3 and 4.

**Table 3:** Outcomes during 12 months after initiation of BUP-XR: commercial/Medicare patients.

Outcomes during 12-month post-index period	Commercial/Medicare							
	All patients	Group 1	Group 2	*p*-value^a^	Group 3	*p*-value^a^	Group 4	*p*-value^a^
	(*n* = 661)	(*n* = 163)	(*n* = 113)		(*n* = 264)		(*n* = 121)	
Post-index MOUD adherence, mean	–	–	–	–	–	–	–	–
BUP-XR PDC during 12 months post-index	0.47	0.93	0.32		0.35		0.25	
BUP-XR PDC during first 6 months post-index	0.63	0.94	0.47		0.59		0.43	
BUP-XR PDC during second 6 months post-index	0.32	0.92	0.17		0.12		0.08	
Overall MOUD PDC during 12 months post-index	0.63	0.95	0.94		0.37		0.50	
Overall MOUD PDC during first 6 months post-index	0.76	0.97	0.94		0.61		0.66	
Overall MOUD PDC during second 6 months post-index	0.51	0.94	0.95		0.13		0.33	
All-cause utilization^b^								
Patients with inpatient admission (excluding detoxification), n (%)	47 (7.1%)	9 (5.5%)	9 (8.0%)	0.463	17 (6.4%)	0.836	12 (9.9%)	0.176
Patients with an ED visit (excluding detoxification), n (%)	210 (31.8%)	40 (24.5%)	33 (29.2%)	0.407	82 (31.1%)	0.154	55 (45.5%)	<0.001
Patients with detoxification, n (%)	105 (15.9%)	8 (4.9%)	16 (14.2%)	0.009	33 (12.5%)	0.011	48 (39.7%)	<0.001
Number of admissions, mean (SD)	0.1 (0.5)	0.1 (0.3)	0.2 (0.8)	0.105	0.1 (0.4)	0.518	0.1 (0.5)	0.128
Number of ED visits, mean (SD)	0.7 (1.4)	0.4 (0.8)	0.8 (1.9)	0.020	0.7 (1.4)	0.016	1.0 (1.6)	<0.001
Number of detoxification events, mean (SD)	0.6 (1.8)	0.1 (0.7)	0.7 (2.5)	0.012	0.3 (1.1)	0.065	1.5 (2.9)	<0.001
Number of outpatient office visits, mean (SD)	12.6 (9.6)	15.4 (9.5)	16.3 (9.9)	0.477	9.6 (8.3)	<0.001	12.1 (9.9)	0.005
Number of other outpatient visits, mean (SD)	35.7 (40.7)	35.7 (32.3)	34.0 (28.9)	0.640	30.8 (40.6)	0.188	47.8 (55.9)	0.023
Number of outpatient pharmacy claims, mean (SD)	37.8 (33.3)	45.2 (39.3)	51.0 (37.1)	0.220	26.3 (25.5)	<0.001	40.5 (27.9)	0.262
All-cause costs^c^, mean (SD)								
Inpatient costs (excluding detoxification)	$2,370 ($14,067)	$1,827 ($9,810)	$5,195 ($28,113)	0.159	$1,419 ($7,263)	0.623	$2,536 ($9,760)	0.546
Outpatient costs (excluding detoxification)	$20,201 ($43,093)	$19,820 ($50,563)	$15,564 ($31,664)	0.428	$17,595 ($34,505)	0.590	$30,727 ($55,408)	0.086
ED visit costs	$940 ($2,590)	$554 ($1,789)	$1,062 ($3,153)	0.091	$894 ($2,304)	0.109	$1,444 ($3,343)	0.004
Outpatient office visit costs	$1,587 ($1,517)	$1,876 ($1,683)	$1,965 ($1,583)	0.658	$1,226 ($1,207)	<0.001	$1,634 ($1,661)	0.230
Other outpatient visit costs	$17,673 ($41,805)	$17,390 ($49,678)	$12,537 ($29,448)	0.353	$15,475 ($33,595)	0.635	$27,649 ($53,493)	0.097
Detoxification costs^d^	$7,945 ($37,502)	$1,015 ($5,667)	$8,404 ($37,423)	0.014	$3,920 ($18,593)	0.053	$25,636 ($72,184)	<0.001
Outpatient pharmacy costs	$12,593 ($15,726)	$22,189 ($16,420)	$12,615 ($21,535)	<0.001	$8,585 ($10,435)	<0.001	$8,390 ($12,104)	<0.001
Total costs	$43,109 ($69,826)	$44,851 ($56,918)	$41,779 ($66,306)	0.681	$31,519 ($47,583)	0.009	$67,290 ($111,743)	0.028
All-cause costs^c^ in separate categories^e^, mean (SD)								
MOUD costs	$11,539 ($8,224)	$22,263 ($6,976)	$9,553 ($4,890)	<0.001	$8,041 ($5,024)	<0.001	$6,577 ($4,121)	<0.001
Non-MOUD costs	$31,570 ($70,032)	$22,588 ($56,588)	$32,225 ($65,126)	0.192	$23,478 ($47,405)	0.861	$60,713 ($112,505)	<0.001
Other utilization^e^								
Number of MOUD claims, mean (SD)	10.3 (6.3)	14.8 (4.6)	16.1 (5.2)	0.030	5.2 (3.1)	<0.001	9.7 (5.3)	<0.001
Patients with urine drug screen, n (%)	512 (77.5%)	134 (82.2%)	100 (88.5%)	0.175	176 (66.7%)	<0.001	102 (84.3%)	0.749
Number of urine drug screens, mean (SD)	7.8 (10.9)	8.3 (8.5)	9.5 (8.6)	0.222	5.4 (10.2)	0.002	10.8 (15.5)	0.074
Patients with psychosocial therapy, n (%)	346 (52.3%)	74 (45.4%)	59 (52.2%)	0.273	133 (50.4%)	0.321	80 (66.1%)	<0.001
Number of psychosocial therapy claims, mean (SD)	12.3 (26.4)	9.0 (18.7)	8.6 (20.9)	0.867	11.1 (24.9)	0.353	23.0 (38.1)	<0.001

BUP-XR, extended-release buprenorphine; ED, emergency department; MOUD, medication for opioid use disorder; PDC, proportion of days covered; SD, standard deviation.

a Based on comparison with Group 1.

b Utilization and costs were evaluated during the 12-month post-index period across four mutually exclusive categories: (1) inpatient admissions excluding detoxification, (2) outpatient services (including ED visits, office visits, other outpatient visits [e.g., imaging, laboratory]) excluding detoxification, (3) detoxification, and (4) outpatient pharmacy.

c Costs were inflated to 2023 US dollars using the medical component of the Consumer Price Index.

d Detoxification costs included costs for the full inpatient admissions with a code indicative of detoxification and costs for detoxification-related outpatient claims.

e Utilization for MOUD, urine drug screens, and psychosocial therapy, which were captured within the four service categories listed above, were also reported separately. MOUD costs were based on outpatient pharmacy-dispensed drugs and outpatient drug administrations, while non-MOUD costs were calculated as total costs minus MOUD costs.

### 3.4 Healthcare utilization

**Figure 2:**
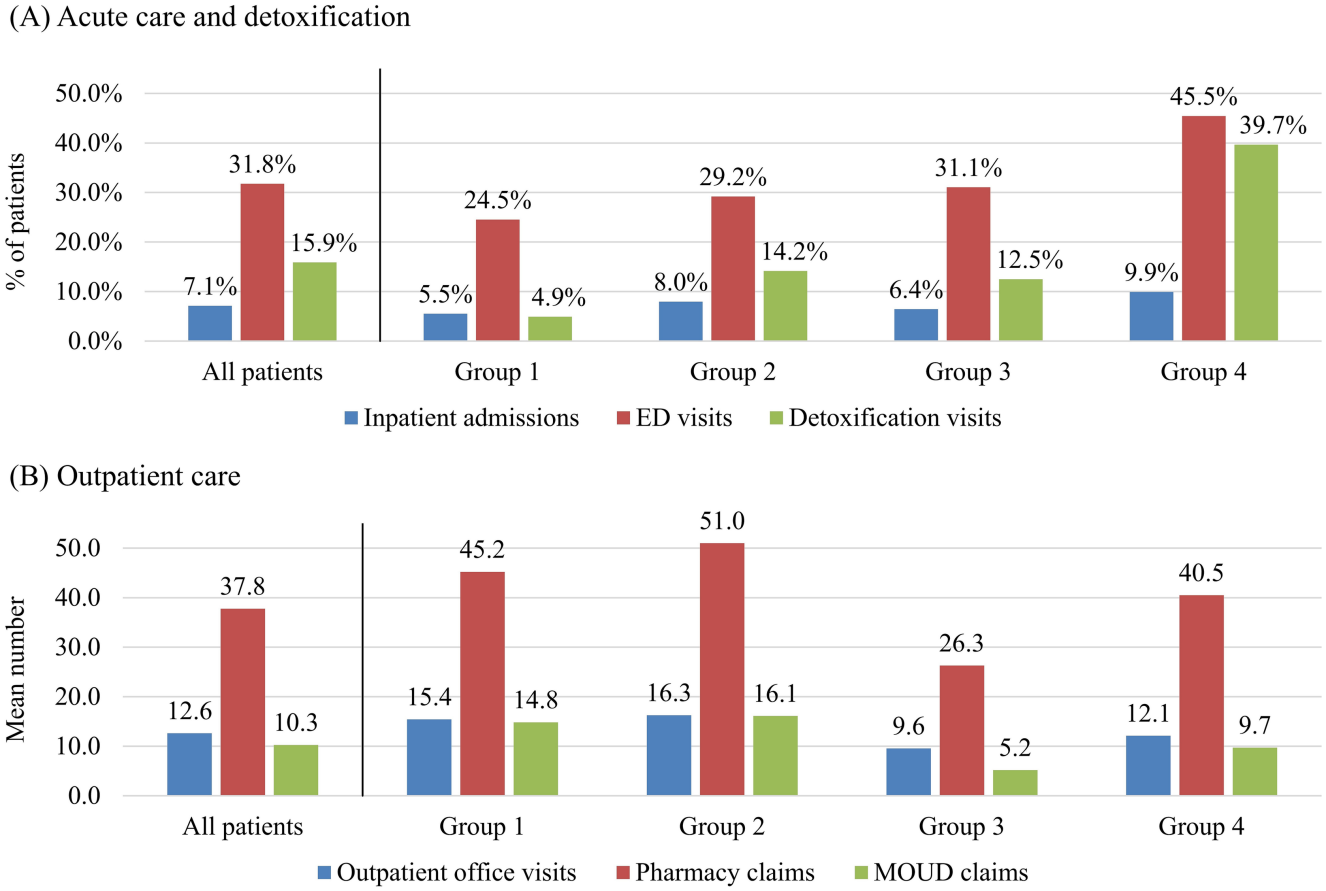
Healthcare utilization for acute care and detoxification **(A)** and outpatient care **(B)** during 12 months after initiation of BUP-XR: commercial/Medicare patients. Commercial/Medicare patients. ED, emergency department; MOUD, medication for opioid use disorder. The proportion of patients with at least one claim is reported for inpatient admissions, ED visits, and detoxification visits. For outpatient office visits, pharmacy claims, and MOUD claims, the mean number of claims is reported, since nearly all patients utilized these services.

All-cause healthcare utilization patterns varied between PDC groups, with adherence influencing levels of both emergent care (e.g., inpatient admissions, ED visits, detoxification visits) and general outpatient care (e.g., office visits, pharmacy claims). Group 1 had the lowest rate of inpatient admissions (5.5%), ED visits (24.5%), and detoxification visits (4.9%) but had high rates of office visits (mean: 15.4) and pharmacy claims (mean: 45.2; Table 3; Figure 2). By contrast, Group 4, with the highest clinical burden, had the most emergent care (9.9% inpatient admissions, 45.5% ED visits, 39.7% detoxification) and lower outpatient care (mean: 12.1 office visits, 40.5 pharmacy claims). Group 3 also showed high emergent utilization (6.4% inpatient admissions, 31.1% ED visits, 12.5% detoxification) but had the lowest outpatient utilization (mean: 9.6 office visits, 26.3 pharmacy claims), suggesting disengagement from care. Finally, Group 2 showed high emergent care use (8.0% inpatient admissions, 29.2% ED visits, 14.2% detoxification), but similar rates of outpatient visits and pharmacy claims to Group 1 (mean: 16.3 office visits, 51.0 pharmacy claims).

Regarding OUD-related care, Groups 1 and 2 had a similarly high number of MOUD claims (14.8 and 16.1 claims, respectively) in comparison to Groups 3 and 4 (5.2 and 9.7 claims, respectively) as expected given they were adherent (Table 3; Figure 2B). Group 4 had the highest psychosocial therapy use (mean: 23.0 claims), possibly reflecting more intensive needs (Table 3). Group 3 had the lowest utilization of urine drug screens (mean: 5.4), indicating less intensive management or possible withdrawal from care, as mentioned above.

### 3.5 Healthcare cost

Consistent with what was observed for healthcare utilization, costs for inpatient admissions, ED visits, and detoxification were higher for other groups in comparison to Group 1. Total healthcare costs during the 12-month post-index period were $44,851 in Group 1, $41,779 in Group 2, $31,519 in Group 3, and $67,290 in Group 4. Non-MOUD costs were the main driver, accounting for 50.4% (Group 1) to 90.2% (Group 4) of total costs (Table 3). Group 1, who were adherent to BUP-XR, incurred the highest MOUD costs ($22,263), yet their non-MOUD costs ($22,588) were comparable to Group 3 ($23,478), and lower than Group 2 ($32,225) and Group 4 ($60,713).

**Figure 3:**
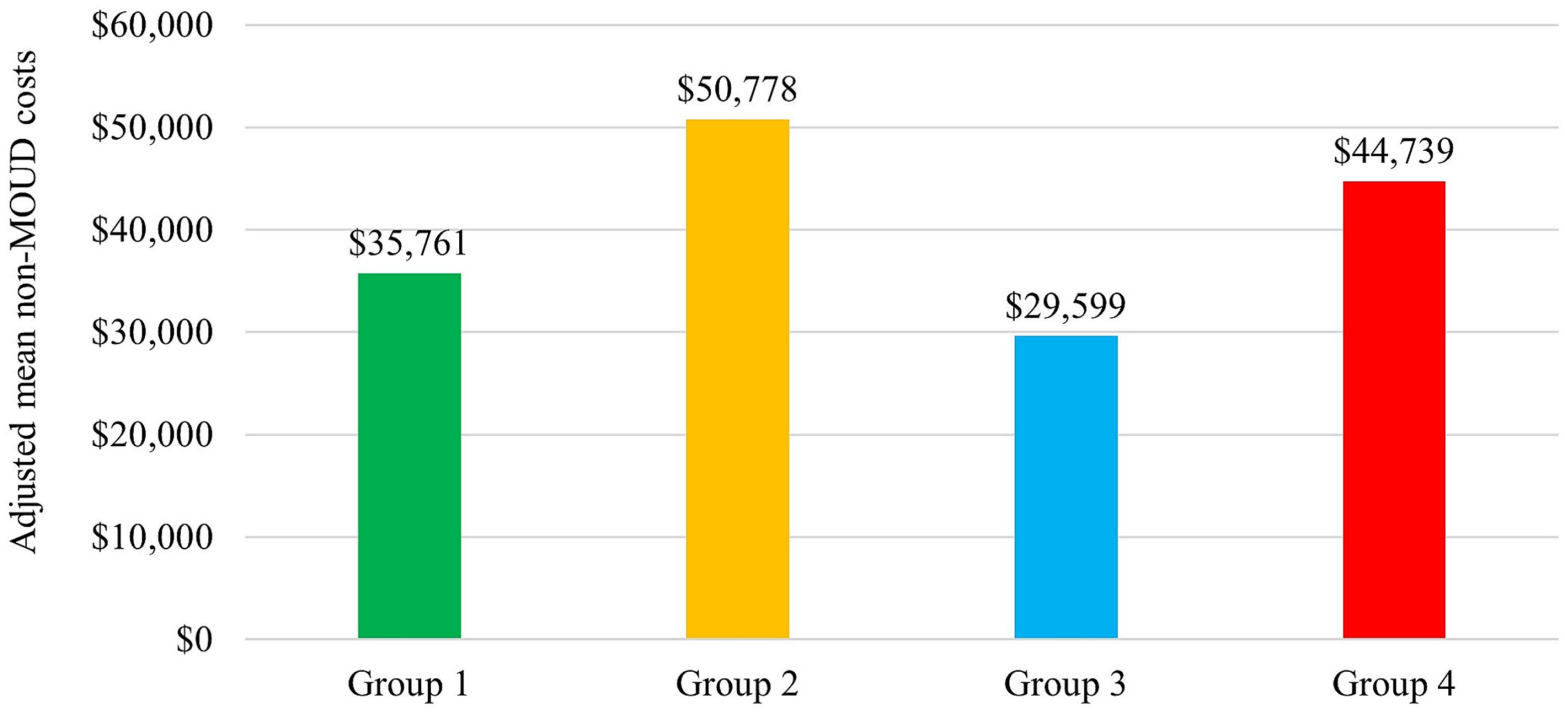
Adjusted mean non-MOUD costs during 12 months after initiation of BUP-XR: commercial/Medicare patients. CI, confidence interval; MOUD, medication for opioid use disorder. Adjusted mean non-MOUD costs (medical and pharmacy costs minus MOUD costs) were reported during the 12-month post-index period, and 95% CIs were $21,286 to $50,236 for Group 1, $28,326 to $73,230 for Group 2, $19,003 to $40,195 for Group 3, and $27,211 to $62,267 for Group 4.

**Figure 4:**
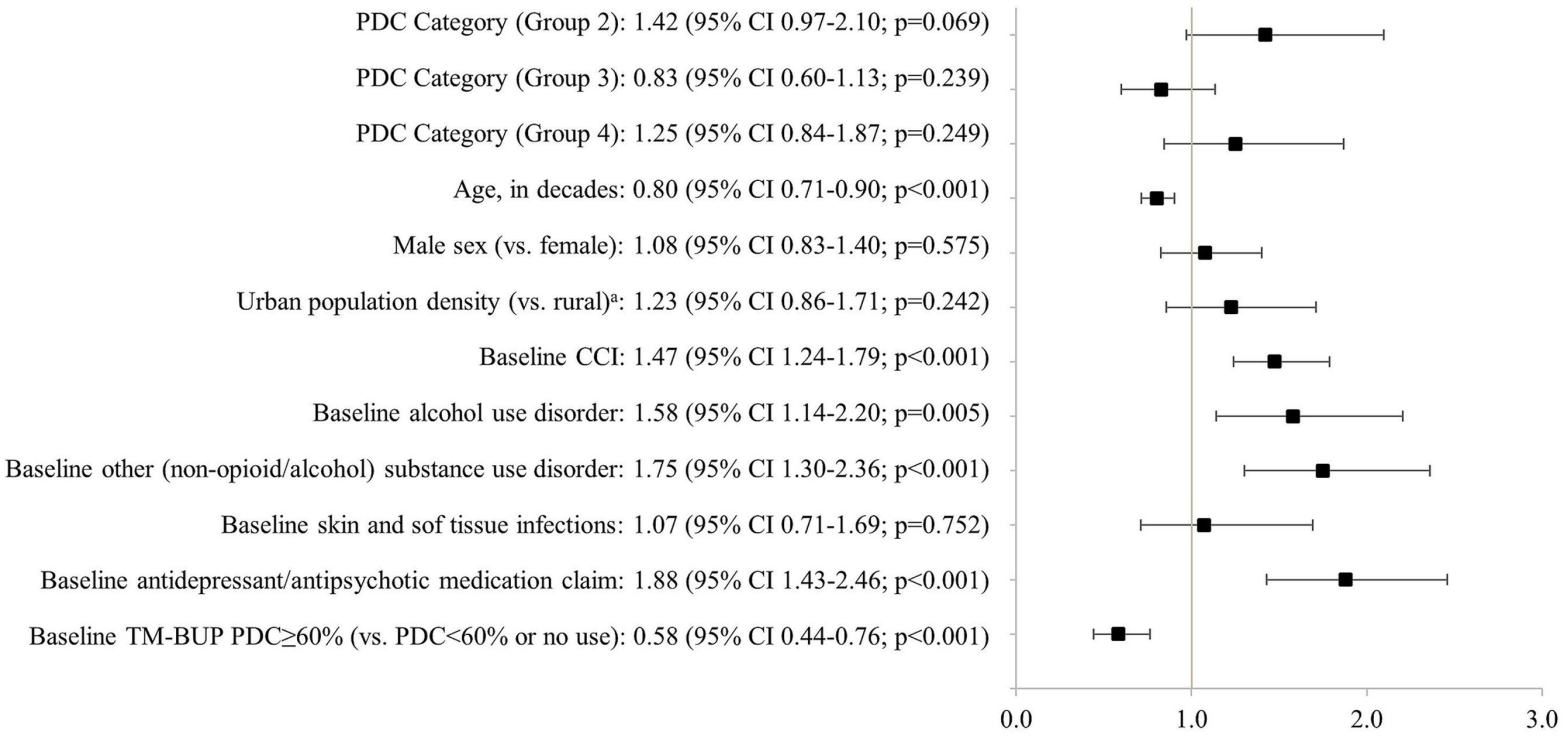
Adjusted cost ratio of mean non-MOUD costs during 12 months after initiation of BUP-XR: commercial/Medicare patients. CCI, Charlson Comorbidity Index; CI, confidence interval; MOUD, medication for opioid use disorder; PDC, proportion of days covered; TM-BUP, transmucosal buprenorphine. Cost ratio for mean non-MOUD costs (medical and pharmacy costs minus MOUD costs) during the 12-month post-index period and 95% confidence interval were reported. Age, sex, payer, and population density were recorded on the index date. Baseline CCI, alcohol use disorder, other (non-opioid/alcohol) substance use disorder, skin and soft tissue infections, and antidepressant/antipsychotic medication claim were evaluated during the 12-month pre-index period plus the index date. Baseline TM-BUP adherence (measured by PDC) was assessed during the 3-month pre-index period. (a) Unknowns were categorized to the urban category (much larger group).

After accounting for differences in baseline characteristics between PDC groups, adjusted mean non-MOUD costs in the 12 months post-index period were $15,017 higher for Group 2 and $8,978 higher for Group 4 relative to Group 1, while costs for Group 3 were the lowest likely due to lack of engagement with care in this group (Group 1: $35,761, Group 2: $50,778, Group 3: $29,599, Group 4: $44,739; Figure 3). Significant predictors of higher non-MOUD costs included younger age, a higher baseline CCI, comorbid alcohol and other non-opioid/substance use disorders, baseline use of antidepressants or antipsychotics, and lack of engagement with prior OUD treatment (no baseline use or PDC < 0.6 with TM-BUP during the three months prior to BUP-XR initiation) (Figure 4). In the sensitivity analysis, which used a less stringent definition of adherence (PDC ≥ 0.6) to define the PDC groups, adjusted mean non-MOUD costs had a consistent pattern with the main analysis, showing considerably higher costs for Group 2 compared to Group 1 and the lowest costs for Group 3 (Group 1: $38,904, Group 2: $48,605, Group 3: $30,321, Group 4: $40,866; Supplementary Figure 2) and the same significant predictors of higher non-MOUD costs (Supplementary Figure 3).

## 4 Discussion

Among commercially insured patients with OUD, this study assessed the proportion of patients who were adherent to both BUP-XR and overall MOUD during the 12 months after initiation of BUP-XR and quantified the association between adherence and healthcare costs. About one quarter (24.7%) of patients were adherent (PDC ≥ 0.8) to BUP-XR (Group 1), 17.1% were adherent to overall MOUD despite being non-adherent to BUP-XR (Group 2), and 58.2% were not adherent (Groups 3 and 4). Previous studies have also shown lack of adherence to MOUD, with only 32 to 43% of patients being adherent (15–17).

Adjusted non-MOUD costs for Group 2, characterized by BUP-XR non-adherence but adherence to overall MOUD, were approximately 42% ($15,017) higher than those adherent to BUP-XR (Group 1). Descriptive analysis showed similar numbers of all-cause office visits, all-cause pharmacy claims, and MOUD claims for Groups 1 and 2 (suggesting similar engagement with their OUD and overall care) yet inpatient admissions, ED visits, and detoxification visits were more common among Group 2 patients (suggesting that BUP-XR adherence may be a key factor in mitigating medical costs for expensive services like acute care and detoxification). From a clinical perspective, patients adherent to BUP-XR maintain stable buprenorphine levels, which are associated with fewer withdrawal symptoms, cravings, and lower rates of relapse. In contrast, patients receiving TM-BUP may struggle with adherence due to the burden of daily dosing. They may also experience gaps in medication coverage due to factors such as care fragmentation (from inconsistent provider relationships or the use of multiple facilities to obtain their TM-BUP prescriptions), pharmacy stock shortages, transportation challenges, or lost or stolen prescriptions. These disruptions can lead to episodes of withdrawal or relapse that require higher-cost care in ED or detoxification centers. Because all patients in Group 2 received at least one BUP-XR injection during the follow-up period (with an average of 2.8 BUP-XR injections within the first 6 months and 3.9 injections during the full 12 months of follow-up), the reported cost difference for Group 1 relative to Group 2 may underestimate the true cost benefit of BUP-XR relative to other MOUD. In fact, therapeutic buprenorphine levels may be maintained for an additional 2 to 5 months following the final BUP-XR injection (19), so patients in Group 2 may have experienced clinical benefit from BUP-XR for a considerable portion of their follow-up.

Group 3, characterized by BUP-XR non-adherence and little to no use of other MOUD, had the lowest adjusted non-MOUD costs, costing about $6,000 less than Group 1 ($29,599 vs. $35,761); however, this likely reflects disengagement from care rather than improved outcomes. Evidence for this includes a significant decline in BUP-XR and overall MOUD adherence from the first half to second half of the follow-up period, low outpatient utilization, and high acute care use (e.g., ED, detoxification). Although consistent patient engagement for regular BUP-XR injections and management increases outpatient healthcare costs associated with preventive medicine (e.g., in the primary care setting), it is a trade-off that helps prevent higher costs for significant acute events like overdose. In clinical practice, patients who disengage from MOUD treatment are at substantially higher risk of return to use of illicit substances and potential overdose. Unfortunately, these same patients often interact less with the healthcare system overall, delaying not only preventive care but also necessary acute care due to concerns about discrimination or stigma associated with their OUD diagnosis. Thus, while high acute care was observed for Group 3 patients, their need for acute care may have been even higher than what was reported. Though a few patients in Group 3 may have tapered off of their BUP-XR treatment, the low rate of office visits relative to the adherent groups (Groups 1 and 2) may suggest that it is less likely that patients had tapered.

Adjusted non-MOUD costs for Group 4 ($44,739), characterized by non-adherence to overall MOUD with use of other MOUD beyond BUP-XR, were considerably lower than unadjusted costs ($60,713), indicating that higher patient complexity (32.2% with alcohol use disorder, 62.0% with other substance use disorder, 80.2% treated with anti-depressants/anti-psychotics, etc.) explained some of the higher unadjusted costs that were reported for this group in comparison to other groups. After adjustment, Group 4 counterintuitively had lower adjusted non-MOUD costs than Group 2 ($44,739 vs. $50,778) despite non-adherence, which may be due to the similar pattern of disengagement from care (e.g., decreasing adherence over time, low outpatient utilization, etc.) seen in Group 3. A patient’s engagement in care and motivation for treatment is not linear. Some patients may have brief initial interactions with treatment followed by rapid disengagement, while others may engage intermittently. For instance, patients may re-engage in treatment following events such as a significant infection or overdose, maintain motivation for a few months, and then disengage again. This intermittent engagement may explain the patterns of non-adherence and treatment fluctuation (e.g., use of BUP-XR at one point and TM-BUP later on) among patients in Group 4. Overall, the potential care disengagement in Group 3 and Group 4 (and therefore, potential cost underestimation among those groups) likely limit direct cost comparisons with the fully engaged Groups 1 and 2. This study therefore highlights that reduced non-MOUD costs are not solely driven by lower clinical needs but also by patient disengagement, emphasizing the critical importance of considering patient engagement when interpreting cost outcomes.

Prior research has established an association between non-adherence to MOUD treatment and higher healthcare costs (14–17). Ronquest et al. examined the relationship between TM-BUP adherence on total healthcare costs among commercially-insured patients with OUD by various PDC categories (<0.2, 0.2- < 0.4, 0.4- < 0.6, 0.6- < 0.8, and ≥0.8), finding that the adjusted total healthcare costs for non-adherent patients were up to 39% higher than adherent patients, depending on extent of non-adherence (15). Ruetsch et al. similarly found 40% higher adjusted total costs over a 12-month period for patients who were non-adherent to TM-BUP relative to adherent patients (16). Unlike other literature that established a cost benefit of adherence versus non-adherence to MOUD, this study found a cost benefit of BUP-XR adherence in comparison to other MOUD adherence (e.g., BUP-XR plus TM-BUP). Together, the findings of this study and previous literature highlight that both adherence level and MOUD treatment selection are important predictors of future healthcare expenditures. Additionally, previous literature showed the same utilization trend as this study (non-adherent patients had more inpatient admissions, ED visits, and detoxification visits and fewer office visits and pharmacy claims) (15, 16), signifying that lower patient engagement in the non-adherent cohorts is not unique to this study and is an important discussion topic for research that associates MOUD adherence with cost outcomes among patients with OUD. Of note, among the two non-adherent groups in this study, Group 4 had higher outpatient office visits (12.1 vs. 9.6) and prescription claims (40.5 vs. 26.3) than Group 3, which may be due to the disproportionately high prevalence of anxiety, depression, and bipolar disorder and other comorbidities in this group. Future research with a nuanced exploration of factors contributing to non-adherence and treatment disengagement would be valuable.

In the supplementary analysis, results using a less stringent definition of adherence (PDC ≥ 0.6) to define the PDC groups were reported. Of note, the proportion of patients who were adherent to BUP-XR (Group 1) increased from 24.7 to 33.3%, representing a slightly higher rate of adherent BUP-XR use. With the less stringent adherence threshold, the adjusted non-MOUD costs for Group 2 (non-adherent to BUP-XR but adherent to overall MOUD) were approximately 25% ($9,701) higher than Group 1 (adherent to BUP-XR), demonstrating a meaningful economic impact of adherence to BUP-XR relative to overall MOUD even with the less stringent adherence definition. It is important to keep in mind that there is no pre-defined duration for maintenance therapy with buprenorphine, so while PDC ≥ 0.8 (main analysis) and ≥0.6 (sensitivity analysis) were used to classify adherent patients in this study, patients with lower PDC may have had treatment success.

This study highlights the clinical and economic benefits of adherence to BUP-XR for OUD, demonstrating that consistent use of BUP-XR helps prevent costly acute medical events like inpatient admissions, ED visits, and detoxification. Patients adherent to BUP-XR had their medical costs reduced by $15,017 per person per year compared to those who were not adherent to BUP-XR but were adherent to other MOUD treatment (e.g., those taking TM-BUP after discontinuing BUP-XR). This suggests that consistent BUP-XR use is more effective than consistent use of other MOUD at containing healthcare expenditures. However, only 24.7% of patients in the study remained adherent to BUP-XR for 12 months post-initiation. Barriers including stigma, discrimination, and fragmented care can lead to treatment disengagement, increasing the risk of relapse, overdose, and acute medical crises that strain healthcare resources. Improving adherence to BUP-XR for OUD requires comprehensive strategies addressing both individual patient barriers and broader systemic challenges. Key interventions may include personalized treatment plans, proactive care coordination to ensure timeliness of subsequent injection visits, the integration of behavioral therapies alongside medication, and improving accessibility to care through telemedicine and community-based services. Public health initiatives should focus on expanding access to evidence-based treatments like BUP-XR, addressing social determinants of health (e.g., socioeconomic status, housing instability, transportation, poverty), and reducing stigma surrounding OUD. Creating a more supportive and non-stigmatizing environment, engaging patients in shared decision-making, providing clear education about treatment options, and ensuring regular follow-up care are crucial for sustained engagement in treatment. As a matter of public health, accessibility and coverage of BUP-XR that prevent disruption in patient care should be prioritized to improve long-term OUD outcomes.

As with any retrospective analyses using administrative claims data, the study is subject to potential errors in data coding and data entry. For example, the use of MOUD may be under-reported because claims might not capture medications provided during inpatient hospital stays or those paid for out of pocket by patients. Second, the generalizability of these results may be less applicable to rural populations (given that 84.7% of the study sample lived in urban areas) or populations with other forms of health coverage (e.g., Tricare for military personnel and their families), uninsured individuals (e.g., those incarcerated), or those experiencing insurance transitions (e.g., switching in and out of Medicaid). While the study population’s private Commercial/Medicare insurance coverage likely reduced financial barriers to BUP-XR and other MOUD, adherence challenges are often driven by factors beyond cost, including psychosocial needs, stigma, and co-occurring mental health disorders. These critical factors should be considered when interpreting the study results. Third, the study period coincided with the COVID-19 pandemic, a time when healthcare access restrictions and systemic disruptions may have influenced the study findings. Fourth, although the assignment of patients into four adherence groups provides a useful framework for analysis, these categories may not fully capture the complexities of patient adherence behaviors, such as fluctuating adherence levels where periods of treatment adherence are interspersed with lapses in treatment. Finally, given the non-interventional study design, potential confounding factors cannot be ruled out. While adjusted analyses controlled for observed differences in baseline characteristics between adherence groups (e.g., baseline CCI, comorbidities and medication use), the study could not account for unmeasurable variables that may influence adherence, healthcare resource utilization and costs. These variables include existing barriers to OUD treatment, distrust and disengagement from care, duration and severity of OUD, motivation to stay on MOUD based on prior treatment history, and social and structural determinants of health. Overall, future research that describes adherence and care engagement patterns over time would be informative to better understand real-world treatment persistence patterns, the factors influencing these patterns, and the association between specific patterns and healthcare utilization and costs.

## 5 Conclusion

Patients with adherence to BUP-XR (PDC ≥ 0.8; BUP-XR product was SUBLOCADE® as no other long-acting injectable buprenorphine products were available during the study period) had a reduction in total healthcare costs (excluding MOUD costs) compared to patients with BUP-XR PDC < 0.8 who maintained adherence to MOUD overall, suggesting that adherence to BUP-XR may have more favorable clinical and economic outcomes than adherence to other MOUD, including TM-BUP. However, only 24.7% of patients initiating BUP-XR had adherence to BUP-XR during the 12 months following treatment initiation. These findings demonstrate the importance of sustaining BUP-XR adherence among patients with OUD in clinical practice, and they highlight a potential opportunity to reduce the need for urgent medical services (and their associated high costs) by increasing the rates of BUP-XR initiation and adherence. While this study showed improved clinical stability (which translates to lower medical costs) for patients with BUP-XR adherence compared to those with BUP-XR non-adherence but adherence to MOUD overall (e.g., those taking TM-BUP after discontinuing BUP-XR), further investigation is needed to explore the benefit of BUP-XR adherence directly compared to other forms of MOUD and to evaluate strategies for improving initiation (e.g., the updated rapid induction dosing for BUP-XR). Understanding whether rapid induction enhances initiation and retention, especially among high-risk patients with OUD and fentanyl exposure, remains a critical area for investigation. Additionally, future research is needed to better understand and quantify how MOUD treatment selection among patients with OUD impacts short-term and long-term patient engagement in both OUD care and overall healthcare.

## Data Availability

The data analyzed in this study is subject to the following licenses/restrictions: the data that support the study findings were provided by Merative. Restrictions apply to the availability of these data, which were used under license for this study and therefore are not publicly available. Requests may be sent to Merative for more information on data availability and licensing. Requests to access these datasets should be directed to https://www.merative.com.
