# Supplementary material for: Association between extended-release buprenorphine adherence and reduced healthcare costs among insured patients with opioid use disorder
Source: Front Public Health. 2026 Mar 5;14:1774410. doi: 10.3389/fpubh.2026.1774410 (PMC13001623; doi:10.3389/fpubh.2026.1774410)
Supplement: Supplementary file 1 [file Data_Sheet_1.docx]

***Supplementary Material***

# Supplement A

## Supplemental Table 1. Definition of PDC groups – Sensitivity Analysis

| **Overall MOUD PDC** | **BUP-XR PDC** | | | | |
| --- | --- | --- | --- | --- | --- |
|  | <0.2 | 0.2 to <0.4 | 0.4 to <0.6 | 0.6 to <0.8 | ≥0.8 |
| <0.2 |  |  |  |  |  |
| 0.2 to <0.4 |  |  |  |  |  |
| 0.4 to <0.6 | Group 4 |  | Group 3 |  |  |
| 0.6 to <0.8 |  |  |  |  |  |
| ≥0.8 |  | Group 2 |  |  | Group 1 |
| **Adherence group definitions** | | | | | |
| Group 1 | Adherent to BUP-XR treatment | | | | |
| Group 2 | Non-adherent to BUP-XR but adherent to overall MOUD | | | | |
| Group 3 | Non-adherent to overall MOUD and primarily treated with BUP-XR | | | | |
| Group 4 | Non-adherent to overall MOUD and not primarily treated with BUP-XR | | | | |

BUP-XR, extended-release buprenorphine; MOUD, medication for opioid use disorder; NTX-XR, extended-release naltrexone; PDC, proportion of days covered; TM-BUP, transmucosal buprenorphine

Adherence was measured by PDC for both BUP-XR and overall MOUD during the 12-month post-index period, categorized as: PDC<0.2, 0.2≤PDC<0.4, 0.4≤PDC<0.6, 0.6≤PDC<0.8, and PDC≥0.8. Overall MOUD included buprenorphine- or naltrexone-based MOUD (BUP-XR, TM-BUP, and NTX-XR). Patients were classified as adherent if they had PDC≥0.8. Patients were classified as being primarily treated with BUP-XR if their BUP-XR PDC and their overall MOUD PDC were both in the same adherence range (eg, if a patient has both BUP-XR PDC and overall MOUD PDC in the 0.4 to <0.6 range then we deduce that BUP-XR was the primary medication utilized, with minimal to no use of other MOUD such as TM-BUP or NTX-XR). PDC groups are defined by the combination of overall MOUD and BUP-XR PDC categories. Group assignments are color-coded as follows: Group 1 (green), Group 2 (yellow), Group 3 (blue), and Group 4 (red). Grey cells indicate combinations of PDC categories that are not possible based on the group definitions.

## Supplemental Table 2. Patient counts, by PDC groups: commercial/Medicare patients

1. Main analysis

|  | **COMMERCIAL/ MEDICARE (n=661)** |
| --- | --- |
| Group 1: Adherent to BUP-XR treatment | 163 (24.7%) |
| Group 2: Non-adherent to BUP-XR but adherent to overall MOUD | 113 (17.1%) |
| Group 3: Non-adherent to overall MOUD and primarily treated with BUP-XR | 264 (39.9%) |
| Group 4: Non-adherent to overall MOUD and not primarily treated with BUP-XR | 121 (18.3%) |

1. Sensitivity analysis

|  | **COMMERCIAL/ MEDICARE (n=661)** |
| --- | --- |
| Group 1: Adherent to BUP-XR treatment | 220 (33.3%) |
| Group 2: Non-adherent to BUP-XR but adherent to overall MOUD | 139 (21.0%) |
| Group 3: Non-adherent to overall MOUD and primarily treated with BUP-XR | 226 (34.2%) |
| Group 4: Non-adherent to overall MOUD and not primarily treated with BUP-XR | 76 (11.5%) |

BUP-XR, extended-release buprenorphine; MOUD, medication for opioid use disorder; NTX-XR, extended-release naltrexone; PDC, proportion of days covered; TM-BUP, transmucosal buprenorphine

Adherence was measured by PDC for both BUP-XR and overall MOUD during the 12-month post-index period, categorized as: PDC<0.2, 0.2≤PDC<0.4, 0.4≤PDC<0.6, 0.6≤PDC<0.8, and PDC≥0.8. Overall MOUD included buprenorphine- or naltrexone-based MOUD (BUP-XR, TM-BUP, and NTX-XR). Patients were classified as adherent if they had PDC≥0.8 (main analysis) or PDC≥0.6 (sensitivity analysis). Patients were classified as being primarily treated with BUP-XR if their overall MOUD PDC and their BUP-XR PDC were both in the same adherence range (eg, if a patient has both overall MOUD PDC and BUP-XR PDC in the 0.4 to <0.6 range then we deduce that BUP-XR was the primary medication utilized, with minimal to no use of other MOUD such as TM-BUP or NTX-XR).

## Supplemental Table 3. Patient counts, by PDC to BUP-XR and all MOUD: commercial/Medicare patients

| **COMMERCIAL/MEDICARE (n=661)** | | | | | | |
| --- | --- | --- | --- | --- | --- | --- |
|  |  | **BUP-XR PDC** | | | | |
|  |  | **<0.2** | **0.2 to <0.4** | **0.4 to <0.6** | **0.6 to <0.8** | **≥0.8** |
| **Overall MOUD PDC** | **<0.2** | 71 (10.7%) |  |  |  |  |
|  | **0.2 to <0.4** | 39 (5.9%) | 77 (11.7%) |  |  |  |
|  | **0.4 to <0.6** | 15 (2.3%) | 22 (3.3%) | 78 (11.8%) |  |  |
|  | **0.6 to <0.8** | 12 (1.8%) | 7 (1.1%) | 26 (3.9%) | 38 (5.8%) |  |
|  | **≥0.8** | 49 (7.4%) | 27 (4.1%) | 18 (2.7%) | 19 (2.9%) | 163 (24.7%) |

BUP-XR, extended-release buprenorphine; MOUD, medication for opioid use disorder; PDC, proportion of days covered

Adherence was measured by PDC for both BUP-XR and overall MOUD during the 12-month post-index period.

## Supplemental Table 4. Demographic and clinical characteristics: commercial/Medicare patients – Sensitivity analysis

| **Baseline demographics and clinical characteristics** | **COMMERCIAL/MEDICARE** | | | | | | | |
| --- | --- | --- | --- | --- | --- | --- | --- | --- |
|  | **All patients** | **Group 1** | **Group 2** | **P-value^a^** | **Group 3** | **P-value^a^** | **Group 4** | **P-value^a^** |
|  | **(n=661)** | **(n=220)** | **(n=139)** |  | **(n=226)** |  | **(n=76)** |  |
| **Demographic characteristics (on index)** |  |  |  |  |  |  |  |  |
| Age, mean (SD) | 37.5 (11.3) | 40.1 (10.7) | 38.8 (11.5) | 0.283 | 36.2 (11.2) | <0.001 | 32.1 (10.7) | <0.001 |
| Age category, n (%) |  |  |  | 0.071 |  | 0.005 |  | <0.001 |
| 18-34 | 274 (41.5%) | 69 (31.4%) | 52 (37.4%) |  | 102 (45.1%) |  | 51 (67.1%) |  |
| 35-44 | 213 (32.2%) | 72 (32.7%) | 51 (36.7%) |  | 74 (32.7%) |  | 16 (21.1%) |  |
| 45-54 | 113 (17.1%) | 56 (25.5%) | 19 (13.7%) |  | 32 (14.2%) |  | 6 (7.9%) |  |
| 55-64 | 59 (8.9%) | 23 (10.5%) | 16 (11.5%) |  | 17 (7.5%) |  | 3 (4.0%) |  |
| 65-74 | 2 (0.3%) | 0 (0.0%) | 1 (0.7%) |  | 1 (0.4%) |  | 0 (0.0%) |  |
| Sex, n (%) |  |  |  | 0.932 |  | 0.929 |  | 0.089 |
| Male | 447 (67.6%) | 151 (68.6%) | 96 (69.1%) |  | 156 (69.0%) |  | 44 (57.9%) |  |
| Female | 214 (32.4%) | 69 (31.4%) | 43 (30.9%) |  | 70 (31.0%) |  | 32 (42.1%) |  |
| Population density, n (%) |  |  |  | 0.776 |  | 0.804 |  | 0.808 |
| Urban | 560 (84.7%) | 186 (84.6%) | 114 (82.0%) |  | 194 (85.8%) |  | 66 (86.8%) |  |
| Rural | 96 (14.5%) | 32 (14.6%) | 24 (17.3%) |  | 31 (13.7%) |  | 9 (11.8%) |  |
| Unknown | 5 (0.8%) | 2 (0.9%) | 1 (0.7%) |  | 1 (0.4%) |  | 1 (1.3%) |  |
| Insurance plan type, n (%) |  |  |  | 0.278 |  | 0.140 |  | 0.857 |
| Comprehensive/Indemnity | 17 (2.6%) | 6 (2.7%) | 6 (4.3%) |  | 1 (0.4%) |  | 4 (5.3%) |  |
| EPO/PPO | 373 (56.4%) | 119 (54.1%) | 85 (61.2%) |  | 128 (56.6%) |  | 41 (54.0%) |  |
| POS/POS with capitation | 71 (10.7%) | 31 (14.1%) | 11 (7.9%) |  | 20 (8.9%) |  | 9 (11.8%) |  |
| HMO | 77 (11.7%) | 23 (10.5%) | 16 (11.5%) |  | 30 (13.3%) |  | 8 (10.5%) |  |
| CDHP/HDHP | 116 (17.6%) | 40 (18.2%) | 19 (13.7%) |  | 44 (19.5%) |  | 13 (17.1%) |  |
| Other/Unknown | 7 (1.1%) | 1 (0.5%) | 2 (1.4%) |  | 3 (1.3%) |  | 1 (1.3%) |  |
| Index year, n (%) |  |  |  | 0.415 |  | 0.340 |  | 0.885 |
| 2019 | 119 (18.0%) | 47 (21.4%) | 25 (18.0%) |  | 34 (15.0%) |  | 13 (17.1%) |  |
| 2020 | 135 (20.4%) | 46 (20.9%) | 24 (17.3%) |  | 48 (21.2%) |  | 17 (22.4%) |  |
| 2021 | 163 (24.7%) | 54 (24.6%) | 32 (23.0%) |  | 57 (25.2%) |  | 20 (26.3%) |  |
| 2022 | 244 (36.9%) | 73 (33.2%) | 58 (41.7%) |  | 87 (38.5%) |  | 26 (34.2%) |  |
| **Baseline clinical characteristics (during 12-month pre-index period plus index date)** |  |  |  |  |  |  |  |  |
| Charlson Comorbidity Index, mean (SD) | 0.4 (0.8) | 0.3 (0.7) | 0.4 (1.0) | 0.798 | 0.3 (0.8) | 0.800 | 0.5 (0.9) | 0.105 |
| Clinical conditions^b^, n (%) |  |  |  |  |  |  |  |  |
| Opioid use disorder^c^ | 585 (88.5%) | 194 (88.2%) | 128 (92.1%) | 0.286 | 195 (86.3%) | 0.573 | 68 (89.5%) | 0.838 |
| Alcohol use disorder^c^ | 146 (22.1%) | 41 (18.6%) | 32 (23.0%) | 0.347 | 48 (21.2%) | 0.554 | 25 (32.9%) | 0.016 |
| Substance use disorder (other than opioids/alcohol)^c^ | 348 (52.7%) | 114 (51.8%) | 68 (48.9%) | 0.665 | 116 (51.3%) | 0.925 | 50 (65.8%) | 0.044 |
| Schizophrenia | 5 (0.8%) | 1 (0.5%) | 1 (0.7%) | 1.000 | 1 (0.4%) | 1.000 | 2 (2.6%) | 0.163 |
| Depression/bipolar disorder | 310 (46.9%) | 104 (47.3%) | 67 (48.2%) | 0.914 | 91 (40.3%) | 0.152 | 48 (63.2%) | 0.023 |
| Generalized anxiety disorder | 344 (52.0%) | 108 (49.1%) | 75 (54.0%) | 0.387 | 110 (48.7%) | 1.000 | 51 (67.1%) | 0.008 |
| HIV/AIDS | 2 (0.3%) | 0 (0.0%) | 1 (0.7%) | 0.387 | 1 (0.4%) | 1.000 | 0 (0.0%) | 1.000 |
| Hepatitis B or C | 37 (5.6%) | 12 (5.5%) | 5 (3.6%) | 0.611 | 12 (5.3%) | 1.000 | 8 (10.5%) | 0.181 |
| Pregnancy | 19 (2.9%) | 2 (0.9%) | 2 (1.4%) | 0.642 | 8 (3.5%) | 0.106 | 7 (9.2%) | 0.001 |
| Endocarditis | 1 (0.2%) | 1 (0.5%) | 0 (0.0%) | 1.000 | 0 (0.0%) | 0.493 | 0 (0.0%) | 1.000 |
| Skin and soft tissue infections | 61 (9.2%) | 17 (7.7%) | 13 (9.4%) | 0.696 | 18 (8.0%) | 1.000 | 13 (17.1%) | 0.027 |
| Chronic pain conditions | 261 (39.5%) | 95 (43.2%) | 54 (38.9%) | 0.443 | 84 (37.2%) | 0.210 | 28 (36.8%) | 0.348 |
| Concomitant medications, n (%) |  |  |  |  |  |  |  |  |
| Narcotic pain medications | 132 (20.0%) | 43 (19.6%) | 30 (21.6%) | 0.687 | 44 (19.5%) | 1.000 | 15 (19.7%) | 1.000 |
| Benzodiazepines | 194 (29.4%) | 52 (23.6%) | 45 (32.4%) | 0.087 | 63 (27.9%) | 0.331 | 34 (44.7%) | <0.001 |
| Sedative/hypnotics | 244 (36.9%) | 64 (29.1%) | 54 (38.9%) | 0.065 | 87 (38.5%) | 0.045 | 39 (51.3%) | <0.001 |
| Antidepressants/antipsychotics | 449 (67.9%) | 155 (70.5%) | 91 (65.5%) | 0.351 | 140 (62.0%) | 0.071 | 63 (82.9%) | 0.035 |
| Antidepressants | 426 (64.5%) | 148 (67.3%) | 88 (63.3%) | 0.494 | 135 (59.7%) | 0.116 | 55 (72.4%) | 0.474 |
| Antipsychotics | 195 (29.5%) | 54 (24.6%) | 38 (27.3%) | 0.620 | 65 (28.8%) | 0.336 | 38 (50.0%) | <0.001 |
| **Baseline MOUD use (during 12-month pre-index period)** |  |  |  |  |  |  |  |  |
| Any use of buprenorphine- or naltrexone-based MOUD^d^, n (%) | 622 (94.1%) | 204 (92.7%) | 139 (100.0%) | <0.001 | 206 (91.2%) | 0.604 | 73 (96.1%) | 0.420 |
| TM-BUP | 621 (94.0%) | 204 (92.7%) | 139 (100.0%) | <0.001 | 205 (90.7%) | 0.494 | 73 (96.1%) | 0.420 |
| NTX-XR | 20 (3.0%) | 5 (2.3%) | 3 (2.2%) | 1.000 | 7 (3.1%) | 0.772 | 5 (6.6%) | 0.131 |
| PDC^e^ for buprenorphine- or naltrexone-based MOUD, mean (SD) | 0.6 (0.4) | 0.6 (0.3) | 0.7 (0.3) | 0.667 | 0.5 (0.4) | <0.001 | 0.4 (0.3) | <0.001 |
| **Baseline TM-BUP use (during 3-month pre-index period), n (%)** |  |  |  | 0.039 |  | 0.010 |  | 0.002 |
| PDC^e^ ≥0.6 | 400 (60.5%) | 145 (65.9%) | 103 (74.1%) |  | 117 (51.8%) |  | 35 (46.1%) |  |
| PDC^e^ <0.6 | 191 (28.9%) | 51 (23.2%) | 31 (22.3%) |  | 75 (33.2%) |  | 34 (44.7%) |  |
| No use of TM-BUP | 70 (10.6%) | 24 (10.9%) | 5 (3.6%) |  | 34 (15.0%) |  | 7 (9.2%) |  |

AIDS, acquired immunodeficiency syndrome; CDHP, consumer-driven health plan; EPO, exclusive provider organization; HDHP, high-deductible health plan; HIV, human immunodeficiency virus; HMO, health maintenance organization; MOUD, medication for opioid use disorder; NTX-XR, extended-release naltrexone; PDC, proportion of days covered; POS, point of service; PPO, preferred provider organization; SD, standard deviation; TM-BUP, transmucosal buprenorphine

^a^Based on comparison with Group 1.

^b^Based on non-diagnostic claims (inpatient or outpatient) with a diagnosis for the given condition.

^c^All substance use disorders include codes for substance abuse and dependence (as applicable).

^d^Based on the patient selection criteria, no patients received extended-release buprenorphine during the 12-month pre-index period or NTX-XR during the 30-day pre-index period.

^e^Calculated as the total days of possession of the medication divided by the length of the reporting period (365 or 90 days). The total days with possession of the medication during the reporting period was calculated regardless of gaps in therapy. For TM-BUP, overlapping days’ supply were appended to the total days’ supply.

## Supplemental Table 5. Outcomes during 12 months after initiation of BUP-XR: commercial/Medicare patients – Sensitivity analysis

|  | **COMMERCIAL/MEDICARE** | | | | | | | |
| --- | --- | --- | --- | --- | --- | --- | --- | --- |
| **Outcomes during 12-month post-index period** | **All patients** | **Group 1** | **Group 2** | **P-value^a^** | **Group 3** | **P-value^a^** | **Group 4** | **P-value^a^** |
|  | **(n=661)** | **(n=220)** | **(n=139)** |  | **(n=226)** |  | **(n=76)** |  |
| **Post-index MOUD adherence, mean** |  |  |  |  |  |  |  |  |
| BUP-XR PDC during 12 months post-index | 0.47 | 0.88 | 0.27 |  | 0.29 |  | 0.19 |  |
| BUP-XR PDC during first 6 months post-index | 0.63 | 0.91 | 0.47 |  | 0.54 |  | 0.34 |  |
| BUP-XR PDC during second 6 months post-index | 0.32 | 0.84 | 0.09 |  | 0.05 |  | 0.04 |  |
| Overall MOUD PDC during 12 months post-index | 0.63 | 0.91 | 0.87 |  | 0.31 |  | 0.38 |  |
| Overall MOUD PDC during first 6 months post-index | 0.76 | 0.94 | 0.91 |  | 0.57 |  | 0.56 |  |
| Overall MOUD PDC during second 6 months post-index | 0.51 | 0.87 | 0.83 |  | 0.06 |  | 0.20 |  |
| **All-cause utilization^b^** |  |  |  |  |  |  |  |  |
| Patients with inpatient admission (excluding detoxification), n (%) | 47 (7.1%) | 16 (7.3%) | 9 (6.5%) | 0.834 | 12 (5.3%) | 0.439 | 10 (13.2%) | 0.156 |
| Patients with an ED visit (excluding detoxification), n (%) | 210 (31.8%) | 62 (28.2%) | 35 (25.2%) | 0.545 | 71 (31.4%) | 0.470 | 42 (55.3%) | <0.001 |
| Patients with detoxification, n (%) | 105 (15.9%) | 15 (6.8%) | 31 (22.3%) | <0.001 | 30 (13.3%) | 0.028 | 29 (38.2%) | <0.001 |
| Number of admissions, mean (SD) | 0.1 (0.5) | 0.1 (0.4) | 0.1 (0.7) | 0.664 | 0.1 (0.3) | 0.355 | 0.2 (0.6) | 0.144 |
| Number of ED visits, mean (SD) | 0.7 (1.4) | 0.6 (1.4) | 0.5 (1.2) | 0.714 | 0.7 (1.3) | 0.628 | 1.2 (1.7) | 0.002 |
| Number of detoxification events, mean (SD) | 0.6 (1.8) | 0.2 (0.7) | 0.9 (2.7) | <0.001 | 0.3 (1.2) | 0.048 | 1.6 (2.9) | <0.001 |
| Number of outpatient office visits, mean (SD) | 12.6 (9.6) | 15.1 (9.7) | 14.9 (9.5) | 0.891 | 9.1 (8.1) | <0.001 | 11.8 (10.2) | 0.014 |
| Number of other outpatient visits, mean (SD) | 35.7 (40.7) | 35.7 (31.9) | 35.7 (33.6) | 0.980 | 31.0 (42.8) | 0.199 | 49.2 (61.6) | 0.015 |
| Number of outpatient pharmacy claims, mean (SD) | 37.8 (33.3) | 44.7 (37.8) | 47.0 (34.5) | 0.551 | 24.8 (24.5) | <0.001 | 39.4 (28.8) | 0.271 |
| **All-cause costs^c^, mean (SD)** |  |  |  |  |  |  |  |  |
| Inpatient costs (excluding detoxification) | $2,370 ($14,067) | $3,103 ($15,344) | $2,990 ($21,045) | 0.953 | $948 ($5,708) | 0.049 | $3,340 ($11,483) | 0.902 |
| Outpatient costs (excluding detoxification) | $20,201 ($43,093) | $20,572 ($48,669) | $15,631 ($21,923) | 0.261 | $17,958 ($36,360) | 0.520 | $34,154 ($65,751) | 0.058 |
| ED visit costs | $940 ($2,590) | $744 ($2,078) | $804 ($2,828) | 0.819 | $915 ($2,364) | 0.420 | $1,828 ($3,739) | 0.002 |
| Outpatient office visit costs | $1,587 ($1,517) | $1,843 ($1,640) | $1,876 ($1,615) | 0.851 | $1,171 ($1,194) | <0.001 | $1,558 ($1,564) | 0.188 |
| Other outpatient visit costs | $17,673 ($41,805) | $17,984 ($47,373) | $12,951 ($21,038) | 0.238 | $15,872 ($35,404) | 0.593 | $30,768 ($63,453) | 0.065 |
| Detoxification costs^d^ | $7,945 ($37,502) | $1,701 ($7,968) | $18,644 ($70,555) | <0.001 | $4,311 ($19,867) | 0.071 | $17,261 ($37,325) | <0.001 |
| Outpatient pharmacy costs | $12,593 ($15,726) | $20,233 ($15,493) | $12,153 ($21,851) | <0.001 | $7,612 ($9,835) | <0.001 | $6,094 ($4,191) | <0.001 |
| Total costs | $43,109 ($69,826) | $45,609 ($56,324) | $49,417 ($93,774) | 0.631 | $30,829 ($50,301) | 0.004 | $60,848 ($94,905) | 0.094 |
| **All-cause costs^c^ in separate categories^e^, mean (SD)** |  |  |  |  |  |  |  |  |
| MOUD costs | $11,539 ($8,224) | $20,778 ($6,642) | $8,567 ($4,129) | <0.001 | $6,661 ($3,858) | <0.001 | $4,731 ($2,209) | <0.001 |
| Non-MOUD costs | $31,570 ($70,032) | $24,831 ($56,138) | $40,850 ($93,869) | 0.044 | $24,169 ($50,154) | 0.896 | $56,117 ($95,547) | <0.001 |
| **Other utilization^e^** |  |  |  |  |  |  |  |  |
| Number of MOUD claims, mean (SD) | 10.3 (6.3) | 14.0 (4.6) | 15.4 (5.6) | 0.010 | 4.4 (2.4) | <0.001 | 7.7 (4.1) | <0.001 |
| Patients with urine drug screen, n (%) | 512 (77.5%) | 175 (79.6%) | 123 (88.5%) | 0.031 | 150 (66.4%) | 0.002 | 64 (84.2%) | 0.405 |
| Number of urine drug screens, mean (SD) | 7.8 (10.9) | 8.0 (8.2) | 10.1 (10.8) | 0.038 | 5.3 (10.8) | 0.003 | 10.4 (16.0) | 0.086 |
| Patients with psychosocial therapy, n (%) | 346 (52.3%) | 105 (47.7%) | 75 (54.0%) | 0.279 | 115 (50.9%) | 0.509 | 51 (67.1%) | 0.005 |
| Number of psychosocial therapy claims, mean (SD) | 12.3 (26.4) | 8.6 (18.0) | 12.3 (25.2) | 0.101 | 11.8 (26.1) | 0.132 | 24.7 (42.2) | <0.001 |

BUP-XR, extended-release buprenorphine; ED, emergency department; MOUD, medication for opioid use disorder; PDC, proportion of days covered; SD, standard deviation

^a^Based on comparison with Group 1.

^b^Utilization and costs were evaluated during the 12-month post-index period across four mutually exclusive categories: 1) inpatient admissions excluding detoxification, 2) outpatient services (including ED visits, office visits, other outpatient visits [eg, imaging, laboratory, etc.]) excluding detoxification, 3) detoxification, and 4) outpatient pharmacy.

^c^Costs were inflated to 2023 US dollars using the medical component of the Consumer Price Index.

^d^Detoxification costs included costs for the full inpatient admissions with a code indicative of detoxification and costs for detoxification-related outpatient claims.

^e^Utilization for MOUD, urine drug screens, and psychosocial therapy, which were captured within the four service categories listed above, were also reported separately. MOUD costs were based on outpatient pharmacy-dispensed drugs and outpatient drug administrations, while non-MOUD costs were calculated as total costs minus MOUD costs.

## Supplemental Figure 1. Healthcare utilization for acute care and detoxification (A) and outpatient care (B) during 12 months after initiation of BUP-XR: commercial/Medicare – Sensitivity analysis

1. Acute care and detoxification
2. Outpatient care

ED, emergency department; MOUD, medication for opioid use disorder

The proportion of patients with at least one claim is reported for inpatient admissions, ED visits, and detoxification visits. For outpatient office visits, pharmacy claims, and MOUD claims, the mean number of claims is reported, since nearly all patients utilized these services.

## Supplemental Figure 2. Adjusted mean non-MOUD costs during 12 months after initiation of BUP-XR: commercial/Medicare patients – Sensitivity analysis

CI, confidence interval; MOUD, medication for opioid use disorder

Adjusted mean non-MOUD costs (medical and pharmacy costs minus MOUD costs) were reported during the 12-month post-index period, and 95% CIs were $23,589 to $54,218 for Group 1, $28,122 to $69,087 for Group 2, $18,853 to $41,790 for Group 3, and $22,318 to $59,413 for Group 4.

## Supplemental Figure 3. Adjusted cost ratio of mean non-MOUD costs during 12 months after initiation of BUP-XR: commercial/Medicare patients – Sensitivity analysis

CCI, Charlson Comorbidity Index; CI, confidence interval; MOUD, medication for opioid use disorder; PDC, proportion of days covered; TM-BUP, transmucosal buprenorphine

Cost ratio for mean non-MOUD costs (medical and pharmacy costs minus MOUD costs) during the 12-month post-index period and 95% confidence interval were reported.

Age, sex, payer, and population density were recorded on the index date. Baseline CCI, alcohol use disorder, other (non-opioid/alcohol) substance use disorder, skin and soft tissue infections, and antidepressant/antipsychotic medication claim were evaluated during the 12-month pre-index period plus the index date. Baseline TM-BUP adherence (measured by PDC) was assessed during the 3-month pre-index period.

^a^Unknowns were categorized to the Urban category (much larger group).

# Supplement B

## Supplemental Figure 4. Patient attrition flow chart: Medicaid patients


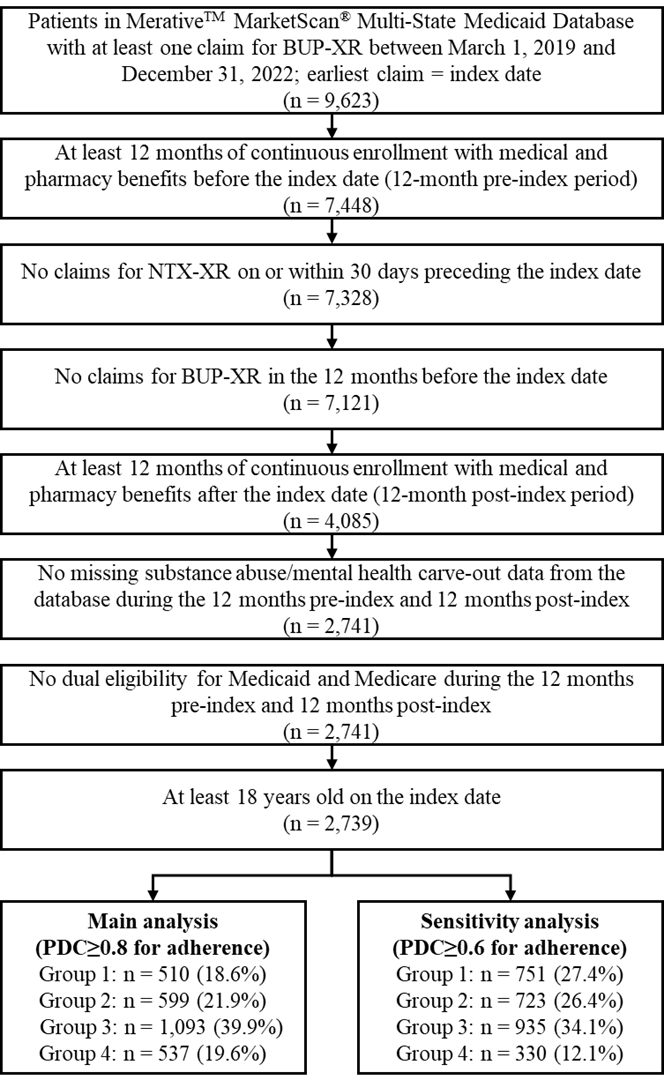


BUP-XR, extended-release buprenorphine; NTX-XR, extended-release naltrexone; PDC, proportion of days covered

## Supplemental Table 6. Patient counts, by PDC groups: Medicaid patients

1. Main analysis

|  | **MEDICAID (n=2,739)** |
| --- | --- |
| Group 1: Adherent to BUP-XR treatment | 510 (18.6%) |
| Group 2: Non-adherent to BUP-XR but adherent to overall MOUD | 599 (21.9%) |
| Group 3: Non-adherent to overall MOUD and primarily treated with BUP-XR | 1,093 (39.9%) |
| Group 4: Non-adherent to overall MOUD and not primarily treated with BUP-XR | 537 (19.6%) |

1. Sensitivity analysis

|  | **MEDICAID (n=2,739)** |
| --- | --- |
| Group 1: Adherent to BUP-XR treatment | 751 (27.4%) |
| Group 2: Non-adherent to BUP-XR but adherent to overall MOUD | 723 (26.4%) |
| Group 3: Non-adherent to overall MOUD and primarily treated with BUP-XR | 935 (34.1%) |
| Group 4: Non-adherent to overall MOUD and not primarily treated with BUP-XR | 330 (12.1%) |

BUP-XR, extended-release buprenorphine; MOUD, medication for opioid use disorder; NTX-XR, extended-release naltrexone; PDC, proportion of days covered; TM-BUP, transmucosal buprenorphine

Adherence was measured by PDC for both BUP-XR and overall MOUD during the 12-month post-index period, categorized as: PDC<0.2, 0.2≤PDC<0.4, 0.4≤PDC<0.6, 0.6≤PDC<0.8, and PDC≥0.8. Overall MOUD included buprenorphine- or naltrexone-based MOUD (BUP-XR, TM-BUP, and NTX-XR). Patients were classified as adherent if they had PDC≥0.8 (main analysis) or PDC≥0.6 (sensitivity analysis). Patients were classified as being primarily treated with BUP-XR if their overall MOUD PDC and their BUP-XR PDC were both in the same adherence range (eg, if a patient has both overall MOUD PDC and BUP-XR PDC in the 0.4 to <0.6 range then we deduce that BUP-XR was the primary medication utilized, with minimal to no use of other MOUD such as TM-BUP or NTX-XR).

## Supplemental Table 7. Patient counts, by PDC to BUP-XR and all MOUD: Medicaid patients

| **MEDICAID (n=2,739)** | | | | | | |
| --- | --- | --- | --- | --- | --- | --- |
|  |  | **BUP-XR PDC** | | | | |
|  |  | **<0.2** | **0.2 to <0.4** | **0.4 to <0.6** | **0.6 to <0.8** | **≥0.8** |
| **Overall MOUD PDC** | **<0.2** | 398 (14.5%) |  |  |  |  |
|  | **0.2 to <0.4** | 156 (5.7%) | 278 (10.2%) |  |  |  |
|  | **0.4 to <0.6** | 101 (3.7%) | 73 (2.7%) | 259 (9.5%) |  |  |
|  | **0.6 to <0.8** | 82 (3.0%) | 39 (1.4%) | 86 (3.1%) | 158 (5.8%) |  |
|  | **≥0.8** | 316 (11.5%) | 121 (4.4%) | 79 (2.9%) | 83 (3.0%) | 510 (18.6%) |

BUP-XR, extended-release buprenorphine; MOUD, medication for opioid use disorder; PDC, proportion of days covered

Adherence was measured by PDC for both BUP-XR and overall MOUD during the 12-month post-index period.

## Supplemental Table 8A. Demographic and clinical characteristics: Medicaid patients – Main analysis

| **Baseline demographics and clinical characteristics** | **MEDICAID** | | | | | | | |  |
| --- | --- | --- | --- | --- | --- | --- | --- | --- | --- |
|  | **All patients** | **Group 1** | **Group 2** | **P-value^a^** | **Group 3** | **P-value^a^** | **Group 4** | **P-value^a^** | |
|  | **(n=2,739)** | **(n=510)** | **(n=599)** |  | **(n=1,093)** |  | **(n=537)** |  |  |
| **Demographic characteristics (on index)** |  |  |  |  |  |  |  |  | |
| Age, mean (SD) | 35.9 (8.1) | 36.5 (7.8) | 36.4 (7.9) | 0.784 | 35.6 (8.3) | 0.030 | 35.3 (7.9) | 0.016 | |
| Age category, n (%) |  |  |  | 0.659 |  | 0.307 |  | 0.108 | |
| 18-34 | 1,330 (48.6%) | 228 (44.7%) | 281 (46.9%) |  | 542 (49.6%) |  | 279 (52.0%) |  | |
| 35-44 | 1,042 (38.0%) | 208 (40.8%) | 232 (38.7%) |  | 408 (37.3%) |  | 194 (36.1%) |  | |
| 45-54 | 280 (10.2%) | 60 (11.8%) | 64 (10.7%) |  | 107 (9.8%) |  | 49 (9.1%) |  | |
| 55-64 | 86 (3.1%) | 14 (2.8%) | 22 (3.7%) |  | 35 (3.2%) |  | 15 (2.8%) |  | |
| 65-74 | 1 (0.0%) | 0 (0.0%) | 0 (0.0%) |  | 1 (0.1%) |  | 0 (0.0%) |  | |
| Sex, n (%) |  |  |  | 0.196 |  | 0.553 |  | 0.015 | |
| Male | 1,334 (48.7%) | 242 (47.5%) | 261 (43.6%) |  | 536 (49.0%) |  | 295 (54.9%) |  | |
| Female | 1,405 (51.3%) | 268 (52.6%) | 338 (56.4%) |  | 557 (51.0%) |  | 242 (45.1%) |  | |
| Population density, n (%) |  |  |  | 0.991 |  | 0.633 |  | 0.053 | |
| Urban | 1,956 (71.4%) | 353 (69.2%) | 416 (69.5%) |  | 782 (71.6%) |  | 405 (75.4%) |  | |
| Rural | 779 (28.4%) | 156 (30.6%) | 182 (30.4%) |  | 309 (28.3%) |  | 132 (24.6%) |  | |
| Unknown | 4 (0.2%) | 1 (0.2%) | 1 (0.2%) |  | 2 (0.2%) |  | 0 (0.0%) |  | |
| Insurance plan type, n (%) |  |  |  | 0.002 |  | <0.001 |  | <0.001 | |
| Comprehensive/Indemnity | 495 (18.1%) | 110 (21.6%) | 115 (19.2%) |  | 193 (17.7%) |  | 77 (14.3%) |  | |
| EPO/PPO | 1 (0.0%) | 0 (0.0%) | 0 (0.0%) |  | 1 (0.1%) |  | 0 (0.0%) |  | |
| POS/POS with capitation | 253 (9.2%) | 77 (15.1%) | 53 (8.9%) |  | 86 (7.9%) |  | 37 (6.9%) |  | |
| HMO | 1,989 (72.6%) | 323 (63.3%) | 431 (72.0%) |  | 812 (74.3%) |  | 423 (78.8%) |  | |
| CDHP/HDHP | 0 (0.0%) | 0 (0.0%) | 0 (0.0%) |  | 0 (0.0%) |  | 0 (0.0%) |  | |
| Other/Unknown | 1 (0.0%) | 0 (0.0%) | 0 (0.0%) |  | 1 (0.1%) |  | 0 (0.0%) |  | |
| Index year, n (%) |  |  |  | <0.001 |  | <0.001 |  | 0.012 | |
| 2019 | 545 (19.9%) | 101 (19.8%) | 157 (26.2%) |  | 179 (16.4%) |  | 108 (20.1%) |  | |
| 2020 | 624 (22.8%) | 100 (19.6%) | 148 (24.7%) |  | 267 (24.4%) |  | 109 (20.3%) |  | |
| 2021 | 1,112 (40.6%) | 189 (37.1%) | 206 (34.4%) |  | 482 (44.1%) |  | 235 (43.8%) |  | |
| 2022 | 458 (16.7%) | 120 (23.5%) | 88 (14.7%) |  | 165 (15.1%) |  | 85 (15.8%) |  | |
| **Baseline clinical characteristics (during 12-month pre-index period plus index date)** |  |  |  |  |  |  |  |  | |
| Charlson Comorbidity Index, mean (SD) | 0.7 (1.3) | 0.6 (1.2) | 0.8 (1.4) | 0.050 | 0.6 (1.3) | 0.643 | 0.7 (1.2) | 0.135 | |
| Clinical conditions^b^, n (%) |  |  |  |  |  |  |  |  | |
| Opioid use disorder^c^ | 2,651 (96.8%) | 488 (95.7%) | 585 (97.7%) | 0.088 | 1,056 (96.6%) | 0.393 | 522 (97.2%) | 0.241 | |
| Alcohol use disorder^c^ | 649 (23.7%) | 100 (19.6%) | 137 (22.9%) | 0.211 | 256 (23.4%) | 0.094 | 156 (29.1%) | <0.001 | |
| Substance use disorder (other than opioids/alcohol)^c^ | 2,302 (84.1%) | 404 (79.2%) | 495 (82.6%) | 0.166 | 928 (84.9%) | 0.005 | 475 (88.5%) | <0.001 | |
| Schizophrenia | 82 (3.0%) | 18 (3.5%) | 19 (3.2%) | 0.741 | 25 (2.3%) | 0.183 | 20 (3.7%) | 1.000 | |
| Depression/bipolar disorder | 1,758 (64.2%) | 331 (64.9%) | 421 (70.3%) | 0.061 | 657 (60.1%) | 0.069 | 349 (65.0%) | 1.000 | |
| Generalized anxiety disorder | 1,701 (62.1%) | 320 (62.8%) | 403 (67.3%) | 0.129 | 619 (56.6%) | 0.022 | 359 (66.9%) | 0.174 | |
| HIV/AIDS | 30 (1.1%) | 5 (1.0%) | 10 (1.7%) | 0.436 | 12 (1.1%) | 1.000 | 3 (0.6%) | 0.496 | |
| Hepatitis B or C | 832 (30.4%) | 132 (25.9%) | 182 (30.4%) | 0.108 | 323 (29.6%) | 0.137 | 195 (36.3%) | <0.001 | |
| Pregnancy | 197 (7.2%) | 36 (7.1%) | 45 (7.5%) | 0.817 | 82 (7.5%) | 0.837 | 34 (6.3%) | 0.711 | |
| Endocarditis | 40 (1.5%) | 10 (2.0%) | 7 (1.2%) | 0.332 | 10 (0.9%) | 0.092 | 13 (2.4%) | 0.677 | |
| Skin and soft tissue infections | 645 (23.6%) | 102 (20.0%) | 140 (23.4%) | 0.189 | 255 (23.3%) | 0.139 | 148 (27.6%) | 0.005 | |
| Chronic pain conditions | 1,089 (39.8%) | 203 (39.8%) | 281 (46.9%) | 0.018 | 386 (35.3%) | 0.085 | 219 (40.8%) | 0.753 | |
| Concomitant medications, n (%) |  |  |  |  |  |  |  |  | |
| Narcotic pain medications | 528 (19.3%) | 92 (18.0%) | 109 (18.2%) | 1.000 | 202 (18.5%) | 0.890 | 125 (23.3%) | 0.040 | |
| Benzodiazepines | 681 (24.9%) | 126 (24.7%) | 177 (29.6%) | 0.079 | 221 (20.2%) | 0.044 | 157 (29.2%) | 0.109 | |
| Sedative/hypnotics | 1,360 (49.7%) | 232 (45.5%) | 319 (53.3%) | 0.011 | 520 (47.6%) | 0.452 | 289 (53.8%) | 0.008 | |
| Antidepressants/antipsychotics | 2,149 (78.5%) | 404 (79.2%) | 480 (80.1%) | 0.709 | 831 (76.0%) | 0.161 | 434 (80.8%) | 0.537 | |
| Antidepressants | 1,983 (72.4%) | 369 (72.4%) | 453 (75.6%) | 0.217 | 761 (69.6%) | 0.290 | 400 (74.5%) | 0.442 | |
| Antipsychotics | 1,136 (41.5%) | 195 (38.2%) | 249 (41.6%) | 0.269 | 431 (39.4%) | 0.660 | 261 (48.6%) | <0.001 | |
| **Baseline MOUD use (during 12-month pre-index period)** |  |  |  |  |  |  |  |  | |
| Any use of buprenorphine- or naltrexone-based MOUD^d^, n (%) | 2,618 (95.6%) | 491 (96.3%) | 596 (99.5%) | <0.001 | 1,017 (93.1%) | 0.012 | 514 (95.7%) | 0.753 | |
| TM-BUP | 2,613 (95.4%) | 491 (96.3%) | 596 (99.5%) | <0.001 | 1,013 (92.7%) | 0.005 | 513 (95.5%) | 0.641 | |
| NTX-XR | 126 (4.6%) | 15 (2.9%) | 27 (4.5%) | 0.207 | 52 (4.8%) | 0.107 | 32 (6.0%) | 0.024 | |
| PDC^e^ for buprenorphine- or naltrexone-based MOUD, mean (SD) | 0.5 (0.3) | 0.6 (0.4) | 0.7 (0.3) | <0.001 | 0.4 (0.3) | <0.001 | 0.5 (0.3) | <0.001 | |
| **Baseline TM-BUP use (during 3-month pre-index period), n (%)** |  |  |  | <0.001 |  | <0.001 |  | <0.001 | |
| PDC^e^ ≥0.6 | 1,563 (57.1%) | 336 (65.9%) | 460 (76.8%) |  | 501 (45.8%) |  | 266 (49.5%) |  | |
| PDC^e^ <0.6 | 937 (34.2%) | 142 (27.8%) | 129 (21.5%) |  | 443 (40.5%) |  | 223 (41.5%) |  | |
| No use of TM-BUP | 239 (8.7%) | 32 (6.3%) | 10 (1.7%) |  | 149 (13.6%) |  | 48 (8.9%) |  | |

AIDS, acquired immunodeficiency syndrome; CDHP, consumer-driven health plan; EPO, exclusive provider organization; HDHP, high-deductible health plan; HIV, human immunodeficiency virus; HMO, health maintenance organization; MOUD, medication for opioid use disorder; NTX-XR, extended-release naltrexone; PDC, proportion of days covered; POS, point of service; PPO, preferred provider organization; SD, standard deviation; TM-BUP, transmucosal buprenorphine

^a^Based on comparison with Group 1.

^b^Based on non-diagnostic claims (inpatient or outpatient) with a diagnosis for the given condition.

^c^All substance use disorders include codes for substance abuse and dependence (as applicable).

^d^Based on the patient selection criteria, no patients received extended-release buprenorphine during the 12-month pre-index period or NTX-XR during the 30-day pre-index period.

^e^Calculated as the total days of possession of the medication divided by the length of the reporting period (365 or 90 days). The total days with possession of the medication during the reporting period was calculated regardless of gaps in therapy. For TM-BUP, overlapping days’ supply were appended to the total days’ supply.

## Supplemental Table 8B. Demographic and clinical characteristics: Medicaid patients – Sensitivity analysis

| **Baseline demographics and clinical characteristics** | **MEDICAID** | | | | | | | |
| --- | --- | --- | --- | --- | --- | --- | --- | --- |
|  | **All patients** | **Group 1** | **Group 2** | **P-value^a^** | **Group 3** | **P-value^a^** | **Group 4** | **P-value^a^** |
|  | **(n=2,739)** | **(n=751)** | **(n=723)** |  | **(n=935)** |  | **(n=330)** |  |
| **Demographic characteristics (on index)** |  |  |  |  |  |  |  |  |
| Age, mean (SD) | 35.9 (8.1) | 36.4 (7.9) | 36.1 (7.9) | 0.439 | 35.4 (8.3) | 0.013 | 35.7 (8.2) | 0.186 |
| Age category, n (%) |  |  |  | 0.598 |  | 0.245 |  | 0.422 |
| 18-34 | 1,330 (48.6%) | 347 (46.2%) | 344 (47.6%) |  | 471 (50.4%) |  | 168 (50.9%) |  |
| 35-44 | 1,042 (38.0%) | 294 (39.2%) | 284 (39.3%) |  | 345 (36.9%) |  | 119 (36.1%) |  |
| 45-54 | 280 (10.2%) | 89 (11.9%) | 71 (9.8%) |  | 88 (9.4%) |  | 32 (9.7%) |  |
| 55-64 | 86 (3.1%) | 21 (2.8%) | 24 (3.3%) |  | 30 (3.2%) |  | 11 (3.3%) |  |
| 65-74 | 1 (0.0%) | 0 (0.0%) | 0 (0.0%) |  | 1 (0.1%) |  | 0 (0.0%) |  |
| Sex, n (%) |  |  |  | 0.487 |  | 0.315 |  | 0.103 |
| Male | 1,334 (48.7%) | 351 (46.7%) | 351 (48.6%) |  | 460 (49.2%) |  | 172 (52.1%) |  |
| Female | 1,405 (51.3%) | 400 (53.3%) | 372 (51.5%) |  | 475 (50.8%) |  | 158 (47.9%) |  |
| Population density, n (%) |  |  |  | 0.832 |  | 0.400 |  | 0.132 |
| Urban | 1,956 (71.4%) | 521 (69.4%) | 512 (70.8%) |  | 675 (72.2%) |  | 248 (75.2%) |  |
| Rural | 779 (28.4%) | 229 (30.5%) | 210 (29.1%) |  | 258 (27.6%) |  | 82 (24.9%) |  |
| Unknown | 4 (0.2%) | 1 (0.1%) | 1 (0.1%) |  | 2 (0.2%) |  | 0 (0.0%) |  |
| Insurance plan type, n (%) |  |  |  | <0.001 |  | <0.001 |  | <0.001 |
| Comprehensive/Indemnity | 495 (18.1%) | 171 (22.8%) | 127 (17.6%) |  | 149 (15.9%) |  | 48 (14.6%) |  |
| EPO/PPO | 1 (0.0%) | 0 (0.0%) | 0 (0.0%) |  | 1 (0.1%) |  | 0 (0.0%) |  |
| POS/POS with capitation | 253 (9.2%) | 98 (13.1%) | 60 (8.3%) |  | 73 (7.8%) |  | 22 (6.7%) |  |
| HMO | 1,989 (72.6%) | 482 (64.2%) | 536 (74.1%) |  | 711 (76.0%) |  | 260 (78.8%) |  |
| CDHP/HDHP | 0 (0.0%) | 0 (0.0%) | 0 (0.0%) |  | 0 (0.0%) |  | 0 (0.0%) |  |
| Other/Unknown | 1 (0.0%) | 0 (0.0%) | 0 (0.0%) |  | 1 (0.1%) |  | 0 (0.0%) |  |
| Index year, n (%) |  |  |  | 0.007 |  | <0.001 |  | 0.114 |
| 2019 | 545 (19.9%) | 154 (20.5%) | 181 (25.0%) |  | 146 (15.6%) |  | 64 (19.4%) |  |
| 2020 | 624 (22.8%) | 159 (21.2%) | 162 (22.4%) |  | 233 (24.9%) |  | 70 (21.2%) |  |
| 2021 | 1,112 (40.6%) | 277 (36.9%) | 272 (37.6%) |  | 420 (44.9%) |  | 143 (43.3%) |  |
| 2022 | 458 (16.7%) | 161 (21.4%) | 108 (14.9%) |  | 136 (14.6%) |  | 53 (16.1%) |  |
| **Baseline clinical characteristics (during 12-month pre-index period plus index date)** |  |  |  |  |  |  |  |  |
| Charlson Comorbidity Index, mean (SD) | 0.7 (1.3) | 0.6 (1.3) | 0.7 (1.3) | 0.173 | 0.6 (1.3) | 0.815 | 0.7 (1.2) | 0.269 |
| Clinical conditions^b^, n (%) |  |  |  |  |  |  |  |  |
| Opioid use disorder^c^ | 2,651 (96.8%) | 722 (96.1%) | 708 (97.9%) | 0.047 | 902 (96.5%) | 0.795 | 319 (96.7%) | 0.730 |
| Alcohol use disorder^c^ | 649 (23.7%) | 159 (21.2%) | 191 (26.4%) | 0.020 | 220 (23.5%) | 0.265 | 79 (23.9%) | 0.339 |
| Substance use disorder (other than opioids/alcohol)^c^ | 2,302 (84.1%) | 601 (80.0%) | 609 (84.2%) | 0.041 | 800 (85.6%) | 0.003 | 292 (88.5%) | <0.001 |
| Schizophrenia | 82 (3.0%) | 24 (3.2%) | 24 (3.3%) | 1.000 | 21 (2.3%) | 0.287 | 13 (3.9%) | 0.586 |
| Depression/bipolar disorder | 1,758 (64.2%) | 485 (64.6%) | 498 (68.9%) | 0.087 | 565 (60.4%) | 0.086 | 210 (63.6%) | 0.783 |
| Generalized anxiety disorder | 1,701 (62.1%) | 467 (62.2%) | 483 (66.8%) | 0.065 | 535 (57.2%) | 0.041 | 216 (65.5%) | 0.338 |
| HIV/AIDS | 30 (1.1%) | 10 (1.3%) | 9 (1.2%) | 1.000 | 8 (0.9%) | 0.353 | 3 (0.9%) | 0.764 |
| Hepatitis B or C | 832 (30.4%) | 199 (26.5%) | 228 (31.5%) | 0.034 | 284 (30.4%) | 0.083 | 121 (36.7%) | <0.001 |
| Pregnancy | 197 (7.2%) | 58 (7.7%) | 47 (6.5%) | 0.418 | 68 (7.3%) | 0.780 | 24 (7.3%) | 0.901 |
| Endocarditis | 40 (1.5%) | 13 (1.7%) | 12 (1.7%) | 1.000 | 9 (1.0%) | 0.197 | 6 (1.8%) | 1.000 |
| Skin and soft tissue infections | 645 (23.6%) | 158 (21.0%) | 180 (24.9%) | 0.083 | 220 (23.5%) | 0.240 | 87 (26.4%) | 0.058 |
| Chronic pain conditions | 1,089 (39.8%) | 296 (39.4%) | 331 (45.8%) | 0.015 | 332 (35.5%) | 0.105 | 130 (39.4%) | 1.000 |
| Concomitant medications, n (%) |  |  |  |  |  |  |  |  |
| Narcotic pain medications | 528 (19.3%) | 136 (18.1%) | 135 (18.7%) | 0.788 | 175 (18.7%) | 0.801 | 82 (24.9%) | 0.013 |
| Benzodiazepines | 681 (24.9%) | 181 (24.1%) | 215 (29.7%) | 0.016 | 189 (20.2%) | 0.058 | 96 (29.1%) | 0.096 |
| Sedative/hypnotics | 1,360 (49.7%) | 334 (44.5%) | 390 (53.9%) | <0.001 | 459 (49.1%) | 0.062 | 177 (53.6%) | 0.007 |
| Antidepressants/antipsychotics | 2,149 (78.5%) | 587 (78.2%) | 579 (80.1%) | 0.370 | 716 (76.6%) | 0.448 | 267 (80.9%) | 0.331 |
| Antidepressants | 1,983 (72.4%) | 541 (72.0%) | 539 (74.6%) | 0.290 | 655 (70.1%) | 0.388 | 248 (75.2%) | 0.299 |
| Antipsychotics | 1,136 (41.5%) | 287 (38.2%) | 322 (44.5%) | 0.015 | 371 (39.7%) | 0.547 | 156 (47.3%) | 0.006 |
| **Baseline MOUD use (during 12-month pre-index period)** |  |  |  |  |  |  |  |  |
| Any use of buprenorphine- or naltrexone-based MOUD^d^, n (%) | 2,618 (95.6%) | 719 (95.7%) | 713 (98.6%) | <0.001 | 871 (93.2%) | 0.026 | 315 (95.5%) | 0.872 |
| TM-BUP | 2,613 (95.4%) | 719 (95.7%) | 713 (98.6%) | <0.001 | 867 (92.7%) | 0.009 | 314 (95.2%) | 0.635 |
| NTX-XR | 126 (4.6%) | 28 (3.7%) | 31 (4.3%) | 0.598 | 44 (4.7%) | 0.335 | 23 (7.0%) | 0.028 |
| PDC^e^ for buprenorphine- or naltrexone-based MOUD, mean (SD) | 0.5 (0.3) | 0.6 (0.3) | 0.6 (0.3) | 0.022 | 0.4 (0.3) | <0.001 | 0.5 (0.3) | <0.001 |
| **Baseline TM-BUP use (during 3-month pre-index period), n (%)** |  |  |  | <0.001 |  | <0.001 |  | <0.001 |
| PDC^e^ ≥0.6 | 1,563 (57.1%) | 485 (64.6%) | 505 (69.9%) |  | 414 (44.3%) |  | 159 (48.2%) |  |
| PDC^e^ <0.6 | 937 (34.2%) | 209 (27.8%) | 194 (26.8%) |  | 394 (42.1%) |  | 140 (42.4%) |  |
| No use of TM-BUP | 239 (8.7%) | 57 (7.6%) | 24 (3.3%) |  | 127 (13.6%) |  | 31 (9.4%) |  |

AIDS, acquired immunodeficiency syndrome; CDHP, consumer-driven health plan; EPO, exclusive provider organization; HDHP, high-deductible health plan; HIV, human immunodeficiency virus; HMO, health maintenance organization; MOUD, medication for opioid use disorder; NTX-XR, extended-release naltrexone; PDC, proportion of days covered; POS, point of service; PPO, preferred provider organization; SD, standard deviation; TM-BUP, transmucosal buprenorphine

^a^Based on comparison with Group 1.

^b^Based on non-diagnostic claims (inpatient or outpatient) with a diagnosis for the given condition.

^c^All substance use disorders include codes for substance abuse and dependence (as applicable).

^d^Based on the patient selection criteria, no patients received extended-release buprenorphine during the 12-month pre-index period or NTX-XR during the 30-day pre-index period.

^e^Calculated as the total days of possession of the medication divided by the length of the reporting period (365 or 90 days). The total days with possession of the medication during the reporting period was calculated regardless of gaps in therapy. For TM-BUP, overlapping days’ supply were appended to the total days’ supply.

## Supplemental Table 9A. Outcomes during 12 months after initiation of BUP-XR: Medicaid patients – Main analysis

|  | **MEDICAID** | | | | | | | |
| --- | --- | --- | --- | --- | --- | --- | --- | --- |
| **Outcomes during 12-month post-index period** | **All patients** | **Group 1** | **Group 2** | **P-value^a^** | **Group 3** | **P-value^a^** | **Group 4** | **P-value^a^** |
|  | **(n=2,739)** | **(n=510)** | **(n=599)** |  | **(n=1,093)** |  | **(n=537)** |  |
| **Post-index MOUD adherence, mean** |  |  |  |  |  |  |  |  |
| BUP-XR PDC during 12 months post-index | 0.41 | 0.93 | 0.28 |  | 0.32 |  | 0.22 |  |
| BUP-XR PDC during first 6 months post-index | 0.56 | 0.94 | 0.45 |  | 0.54 |  | 0.38 |  |
| BUP-XR PDC during second 6 months post-index | 0.25 | 0.92 | 0.12 |  | 0.11 |  | 0.06 |  |
| Overall MOUD PDC during 12 months post-index | 0.62 | 0.95 | 0.95 |  | 0.35 |  | 0.51 |  |
| Overall MOUD PDC during first 6 months post-index | 0.74 | 0.96 | 0.95 |  | 0.57 |  | 0.64 |  |
| Overall MOUD PDC during second 6 months post-index | 0.51 | 0.94 | 0.94 |  | 0.13 |  | 0.38 |  |
| **All-cause utilization^b^** |  |  |  |  |  |  |  |  |
| Patients with inpatient admission (excluding detoxification), n (%) | 359 (13.1%) | 32 (6.3%) | 88 (14.7%) | <0.001 | 147 (13.5%) | <0.001 | 92 (17.1%) | <0.001 |
| Patients with an ED visit (excluding detoxification), n (%) | 1,566 (57.2%) | 241 (47.3%) | 344 (57.4%) | <0.001 | 605 (55.4%) | 0.003 | 376 (70.0%) | <0.001 |
| Patients with detoxification, n (%) | 530 (19.4%) | 25 (4.9%) | 84 (14.0%) | <0.001 | 220 (20.1%) | <0.001 | 201 (37.4%) | <0.001 |
| Number of admissions, mean (SD) | 0.3 (0.9) | 0.1 (0.6) | 0.3 (0.9) | <0.001 | 0.3 (1.0) | 0.002 | 0.4 (1.1) | <0.001 |
| Number of ED visits, mean (SD) | 1.6 (2.9) | 1.1 (1.8) | 1.7 (2.9) | <0.001 | 1.6 (3.3) | 0.001 | 2.1 (2.9) | <0.001 |
| Number of detoxification events, mean (SD) | 1.6 (5.5) | 0.2 (1.3) | 1.2 (5.1) | <0.001 | 1.3 (4.0) | <0.001 | 4.2 (9.1) | <0.001 |
| Number of outpatient office visits, mean (SD) | 18.5 (15.4) | 22.8 (15.4) | 27.5 (18.5) | <0.001 | 11.8 (10.6) | <0.001 | 17.7 (13.5) | <0.001 |
| Number of other outpatient visits, mean (SD) | 89.0 (109.9) | 84.1 (115.1) | 104.0 (117.9) | 0.005 | 75.0 (100.7) | 0.109 | 105.2 (109.5) | 0.002 |
| Number of outpatient pharmacy claims, mean (SD) | 46.4 (41.2) | 51.0 (39.6) | 71.6 (50.0) | <0.001 | 30.1 (31.0) | <0.001 | 47.2 (33.8) | 0.089 |
| **Other utilization^c^** |  |  |  |  |  |  |  |  |
| Number of MOUD claims, mean (SD) | 13.9 (13.3) | 14.8 (4.2) | 25.9 (19.1) | <0.001 | 5.7 (4.0) | <0.001 | 15.9 (11.9) | 0.042 |
| Patients with urine drug screen, n (%) | 2,558 (93.4%) | 488 (95.7%) | 578 (96.5%) | 0.534 | 977 (89.4%) | <0.001 | 515 (95.9%) | 0.879 |
| Number of urine drug screens, mean (SD) | 15.2 (16.4) | 15.9 (16.7) | 21.3 (18.7) | <0.001 | 10.3 (13.3) | <0.001 | 17.8 (16.2) | 0.066 |
| Patients with psychosocial therapy, n (%) | 2,206 (80.5%) | 379 (74.3%) | 488 (81.5%) | 0.004 | 877 (80.2%) | 0.009 | 462 (86.0%) | <0.001 |
| Number of psychosocial therapy claims, mean (SD) | 37.9 (55.8) | 33.6 (59.0) | 40.4 (57.7) | 0.053 | 34.7 (53.2) | 0.707 | 45.8 (55.0) | <0.001 |

BUP-XR, extended-release buprenorphine; ED, emergency department; MOUD, medication for opioid use disorder; PDC, proportion of days covered; SD, standard deviation

^a^Based on comparison with Group 1.

^b^Utilization was evaluated during the 12-month post-index period across four mutually exclusive categories: 1) inpatient admissions excluding detoxification, 2) outpatient services (including ED visits, office visits, other outpatient visits [eg, imaging, laboratory, etc.]) excluding detoxification, 3) detoxification, and 4) outpatient pharmacy.

^c^Utilization for MOUD, urine drug screens, and psychosocial therapy, which were captured within the four service categories listed, were also reported separately. MOUD utilization was based on outpatient pharmacy-dispensed drugs and outpatient drug administrations.

## Supplemental Table 9B. Outcomes during 12 months after initiation of BUP-XR: Medicaid patients – Sensitivity analysis

|  | **MEDICAID** | | | | | | | |
| --- | --- | --- | --- | --- | --- | --- | --- | --- |
| **Outcomes during 12-month post-index period** | **All patients** | **Group 1** | **Group 2** | **P-value^a^** | **Group 3** | **P-value^a^** | **Group 4** | **P-value^a^** |
|  | **(n=2,739)** | **(n=751)** | **(n=723)** |  | **(n=935)** |  | **(n=330)** |  |
| **Post-index MOUD adherence, mean** |  |  |  |  |  |  |  |  |
| BUP-XR PDC during 12 months post-index | 0.41 | 0.86 | 0.24 |  | 0.26 |  | 0.16 |  |
| BUP-XR PDC during first 6 months post-index | 0.56 | 0.91 | 0.42 |  | 0.49 |  | 0.31 |  |
| BUP-XR PDC during second 6 months post-index | 0.25 | 0.80 | 0.06 |  | 0.04 |  | 0.02 |  |
| Overall MOUD PDC during 12 months post-index | 0.62 | 0.90 | 0.88 |  | 0.28 |  | 0.39 |  |
| Overall MOUD PDC during first 6 months post-index | 0.74 | 0.94 | 0.91 |  | 0.52 |  | 0.54 |  |
| Overall MOUD PDC during second 6 months post-index | 0.51 | 0.86 | 0.84 |  | 0.06 |  | 0.25 |  |
| **All-cause utilization^b^** |  |  |  |  |  |  |  |  |
| Patients with inpatient admission (excluding detoxification), n (%) | 359 (13.1%) | 64 (8.5%) | 106 (14.7%) | <0.001 | 127 (13.6%) | 0.001 | 62 (18.8%) | <0.001 |
| Patients with an ED visit (excluding detoxification), n (%) | 1,566 (57.2%) | 357 (47.5%) | 446 (61.7%) | <0.001 | 526 (56.3%) | <0.001 | 237 (71.8%) | <0.001 |
| Patients with detoxification, n (%) | 530 (19.4%) | 56 (7.5%) | 152 (21.0%) | <0.001 | 199 (21.3%) | <0.001 | 123 (37.3%) | <0.001 |
| Number of admissions, mean (SD) | 0.3 (0.9) | 0.2 (0.7) | 0.3 (0.9) | 0.006 | 0.3 (1.0) | 0.024 | 0.4 (1.1) | <0.001 |
| Number of ED visits, mean (SD) | 1.6 (2.9) | 1.1 (1.8) | 1.8 (3.0) | <0.001 | 1.6 (3.5) | <0.001 | 2.1 (2.7) | <0.001 |
| Number of detoxification events, mean (SD) | 1.6 (5.5) | 0.4 (1.9) | 2.1 (6.5) | <0.001 | 1.4 (4.2) | <0.001 | 4.1 (9.4) | <0.001 |
| Number of outpatient office visits, mean (SD) | 18.5 (15.4) | 22.0 (14.7) | 26.0 (18.0) | <0.001 | 10.8 (10.0) | <0.001 | 15.7 (13.0) | <0.001 |
| Number of other outpatient visits, mean (SD) | 89.0 (109.9) | 89.4 (118.5) | 105.8 (113.2) | 0.007 | 72.7 (99.0) | 0.002 | 97.3 (105.2) | 0.296 |
| Number of outpatient pharmacy claims, mean (SD) | 46.4 (41.2) | 49.8 (39.2) | 67.6 (47.2) | <0.001 | 28.4 (30.2) | <0.001 | 43.3 (34.3) | 0.010 |
| **Other utilization^c^** |  |  |  |  |  |  |  |  |
| Number of MOUD claims, mean (SD) | 13.9 (13.3) | 14.3 (4.9) | 25.0 (18.2) | <0.001 | 5.0 (3.7) | <0.001 | 13.7 (12.5) | 0.276 |
| Patients with urine drug screen, n (%) | 2,558 (93.4%) | 711 (94.7%) | 699 (96.7%) | 0.073 | 830 (88.8%) | <0.001 | 318 (96.4%) | 0.281 |
| Number of urine drug screens, mean (SD) | 15.2 (16.4) | 15.6 (16.8) | 21.2 (17.9) | <0.001 | 10.0 (13.5) | <0.001 | 15.8 (15.3) | 0.853 |
| Patients with psychosocial therapy, n (%) | 2,206 (80.5%) | 568 (75.6%) | 594 (82.2%) | 0.002 | 754 (80.6%) | 0.015 | 290 (87.9%) | <0.001 |
| Number of psychosocial therapy claims, mean (SD) | 37.9 (55.8) | 37.0 (60.7) | 42.9 (56.5) | 0.054 | 33.9 (52.5) | 0.253 | 40.4 (50.5) | 0.387 |

BUP-XR, extended-release buprenorphine; ED, emergency department; MOUD, medication for opioid use disorder; PDC, proportion of days covered; SD, standard deviation

^a^Based on comparison with Group 1.

^b^Utilization was evaluated during the 12-month post-index period across four mutually exclusive categories: 1) inpatient admissions excluding detoxification, 2) outpatient services (including ED visits, office visits, other outpatient visits [eg, imaging, laboratory, etc.]) excluding detoxification, 3) detoxification, and 4) outpatient pharmacy.

^c^Utilization for MOUD, urine drug screens, and psychosocial therapy, which were captured within the four service categories listed, were also reported separately. MOUD utilization was based on outpatient pharmacy-dispensed drugs and outpatient drug administrations.

## Supplemental Figure 5A. Healthcare utilization for acute care and detoxification (A) and outpatient care (B) during 12 months after initiation of BUP-XR: Medicaid patients – Main analysis

1. Acute care and detoxification
2. Outpatient care

ED, emergency department; MOUD, medication for opioid use disorder

The proportion of patients with at least one claim is reported for inpatient admissions, ED visits, and detoxification visits. For outpatient office visits, pharmacy claims, and MOUD claims, the mean number of claims is reported, since nearly all patients utilized these services.

## Supplemental Figure 5B. Healthcare utilization for acute care and detoxification (A) and outpatient care (B) during 12 months after initiation of BUP-XR: Medicaid patients – Sensitivity analysis

1. Acute care and detoxification
2. Outpatient care

ED, emergency department; MOUD, medication for opioid use disorder

The proportion of patients with at least one claim is reported for inpatient admissions, ED visits, and detoxification visits. For outpatient office visits, pharmacy claims, and MOUD claims, the mean number of claims is reported, since nearly all patients utilized these services.
